# Molecular engineering of indenoindene-3-ethylrodanine acceptors with A2-A1-D-A1-A2 architecture for promising fullerene-free organic solar cells

**DOI:** 10.1038/s41598-021-99308-7

**Published:** 2021-10-13

**Authors:** Muhammad Khalid, Momina, Muhammad Imran, Muhammad Fayyaz ur Rehman, Ataualpa Albert Carmo Braga, Muhammad Safwan Akram

**Affiliations:** 1 Department of Chemistry, Khawaja Fareed University of Engineering and Information Technology, Rahim Yar Khan, 64200 Pakistan; 2 Department of Chemistry, Faculty of Science, King Khalid University, P.O. Box 9004, Abha, 61413 Saudi Arabia; 3 Institute of Chemistry, University of Sargodha, Sargodha, 40100 Pakistan; 4 Departamento de Química Fundamental, Instituto de Química, Universidade de São Paulo, Av. Prof. LineuPrestes 748, São Paulo, 05508-000 Brazil; 5 School of Health and Life Sciences, Teesside University, Middlesbrough, TS1 3BX UK; 6 National Horizons Centre, Teesside University, Darlington, DL1 1HG UK

**Keywords:** Materials for optics, Energy

## Abstract

Considering the increased demand and potential of photovoltaic devices in clean, renewable electrical and hi-tech applications, non-fullerene acceptor (NFA) chromophores have gained significant attention. Herein, six novel NFA molecules **IBRD1**–**IBRD6** have been designed by structural modification of the terminal moieties from experimentally synthesized A2-A1-D-A1-A2 architecture **IBR** for better integration in organic solar cells (OSCs). To exploit the electronic, photophysical and photovoltaic behavior, density functional theory/time dependent-density functional theory (DFT/TD-DFT) computations were performed at M06/6-311G(*d*,*p*) functional. The geometry, electrical and optical properties of the designed acceptor molecules were compared with reported **IBR** architecture. Interestingly, a reduction in bandgap (2.528–2.126 eV), with a broader absorption spectrum, was studied in **IBR** derivatives (2.734 eV). Additionally, frontier molecular orbital findings revealed an excellent transfer of charge from donor to terminal acceptors and the central indenoindene-core was considered responsible for the charge transfer. Among all the chromophores, **IBRD3** manifested the lowest energy gap (2.126 eV) with higher *λ*_*max*_ at 734 and 745 nm in gaseous phase and solvent (chloroform), respectively due to the strong electron-withdrawing effect of five end-capped cyano groups present on the terminal acceptor. The transition density matrix map revealed an excellent charge transfer from donor to terminal acceptors. Further, to investigate the charge transfer and open-circuit voltage (*V*_*oc*_), **PBDBT** donor polymer was blended with acceptor chromophores, and a significant *V*_*oc*_ (0.696–1.854 V) was observed. Intriguingly, all compounds exhibited lower reorganization and binding energy with a higher exciton dissociation in an excited state. This investigation indicates that these designed chromophores can serve as excellent electron acceptor molecules in organic solar cells (OSCs) that make them attractive candidates for the development of scalable and inexpensive optoelectronic devices.

## Introduction

Solar energy is the most poised amongst the renewable sources to avert the climate crisis^1,2^. Until now Silicon-based solar cells (SCs) have been used frequently due to their low toxicity, thermal stability, and comparatively impressive power conversion efficiency (PCE); but suffer from certain drawbacks, including the high cost of production, heavy weight (20–30 kg m^−2^), rigidity, as well as defined and unalterable HOMO–LUMO levels^3^. To overcome these drawbacks, significant research effort has been put into more flexible, lightweight (0.5 kg m^−2^) organic solar cells (OSCs)^4^. These show great promise but traditionally suffered from low efficiencies. OSCs are generally bulk heterojunction (BHJ) units where absorption layers blend donor and acceptor molecules. Over the last 2 decades, cell efficiencies have improved from 2 to 18% and it has been possible due to use of fullerenes as electron-accepting moieties^5^. PCE of OSCs containing fullerene and their derivatives like PC61BM or PC71BM is found to be 11–12%, which is comparatively good electron conduction owing to their deep-lying LUMO levels. But fullerene acceptors suffer from the inability to harvest light as their absorption spectrum is poorly matched to the solar spectrum. This is further complicated by low photostability, diffusion into other layers, and lack of tunability^6–11^. This has given a way to research into non-fullerene acceptors (NFAs), particularly small molecules offering electron affinity tuneability and better-suited absorption spectra to capture sunlight^6,7,12–15^. Non-fullerene (NF) solar cells also termed as ‘all polymer’ or fullerene-free solar cells^16^ are considered as next generation OSCs^17^. Fused ring electron acceptors (FREAs) is an emergent class that absorbs visible to near-infrared (NIR) very well^6,12,18–24^ and possess better PCE values^25^, high thermic constancy^26^, and good stability in comparison to other NFAs^27^. In the last decade, there has been a move to design better photovoltaic materials and fine-tune their optoelectronic characteristics utilizing various FREAs, such as star molecule^28^, linear geometric molecules^29^ and X-shaped donor molecule^30^ etc. FREAs are usually A-D-A type chromophores where donor forms the central core, while electron acceptors act as pendants where they play the role of side end-capped groups which can be modified to tune the optoelectronic properties of investigated compounds^13–15,31–34^. This arrangement has been shown to be effective to build up highly desirable optoelectronic materials and the prediction of their electronic characteristics prior to synthesis. The properties of NFAs in OSCs inspired us to use recently synthesized A2-A1-D-A1-A2 type efficient NFA as reference chromophore, shortened as **IBR** synthesized by Zulfiqar et al. with an indenoindene core^35^ and 3-ethylrodanine end-capped acceptor. The indenoindene core in **IBR** enhanced the electron transportation due to extended π-electron conjugation. One *sp*^3^ carbon bridge and conjugated 14π-electrons in indenoindene allows it for tuning crystallinity, energy levels, absorption and solubility. This core has proven very effective in developing photovoltaic materials^36^. Li et al. showed that weak electron-withdrawing units play a vital role in tuning charge transport properties and energy level tuning. Thus, we planned to replace 3-ethylrodanine of **IBR** with different compounds with varying strength of electron removal in combination with indenoindene core to design novel photovoltaic molecules. Further, in the designed compounds, 2-butyloctyl is replaced by a methoxy group taking into account the computational cost. To the best of our information, the photovoltaic investigation of designed compounds (**IBRD1**–**IBRD6**) is unreported. Therefore, for the first time, we reported maximum absorption (*λ*_*max*_), frontier molecular orbital (FMOs), density of states (DOS) analysis, open-circuit voltage (*V*_*oc*_), reorganization energies and transition density matrix (TDM) heat maps of **IBRD1**–**IBRD6** chromophores. Above mentioned properties of designed compounds have been compared with **IBR** to evaluate the performance of end-capped acceptor units. This theoretical insight should offer better design of photovoltaic materials to be used in the OSC applications.

## Methods

**Figure 1:**
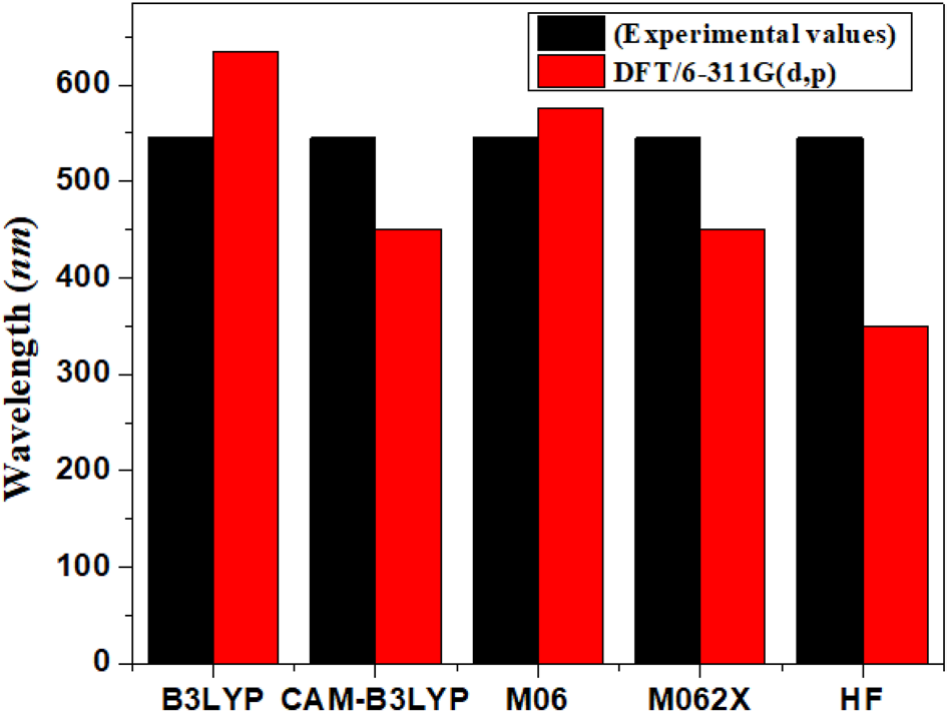
Graphical representation of comparison between experimentally and calculated UV–Vis results of **IBR** at four DFT based functionals and Hartree Fock method (HF) in solvent (CHCl_3_) by utilizing origin 8.5 version (https://www.originlab.com/). All out put files of entitled compounds were accomplished by Gaussian 09 version D.01 (https://gaussian.com/g09citation/).

All the computations for the present work were implemented using Gaussian version 09 software^37^, and calculations were examined through GaussView version 5^38^. First of all, for optimization of geometrical parameters of **IBR**, theoretical calculations were performed at various functionals such as B3LYP^39^, CAM-B3LYP^40^, M06^34^, Hartree Fock method (HF)^41^ and M062X^42^ with a 6-311G(*d*,*p*) basis set. Furthermore, UV–visible investigations for **IBR** was performed at the aforementioned functionals and basis set in chloroform. At the M06 Level, UV–visible findings exhibited an excellent agreement with experimental values (Fig. 1). After the selection of M06 functional, all the derivatives were optimized at this level of theory. To investigate the structure–property relationship and optoelectronic properties of OSCs, absorption spectra, frontier molecular orbital analysis (FMOs), the density of states (DOS), reorganization energy (RE), transition density matrices (TDM), and open-circuit voltage (*V*_*oc*_) were investigated at M06/6-311g(*d*,*p*) level. Moreover, the charge transfer phenomena for the complexes (**PBDBT:IBRD3** and **PBDBT:IBR-IBRD6**) was investigated at M06 and $\omega$ B97XD with 3-21G basis set. Frequently, the functional ($\omega$ B97XD) was utilized to explore the dispersion forces^43^. Subsequently, the charge transformation is significantly observed from donor to acceptor in **PBDBT:IBRD3** and **PBDBT:IBRD6** at $\omega$ B97XD/3-21G functional (see Figure S2) as was reported at M06/3-21G level. Various software, including Multiwfn version 3.8^44^, PyMOlyze version 2.0^45^, Avogadro version 1.2.0n^46^, Gaussview version 5.0^38^ and Chemcraft build 595b^47^ were used for data analysis.

## Results and discussion

**Figure 2:**
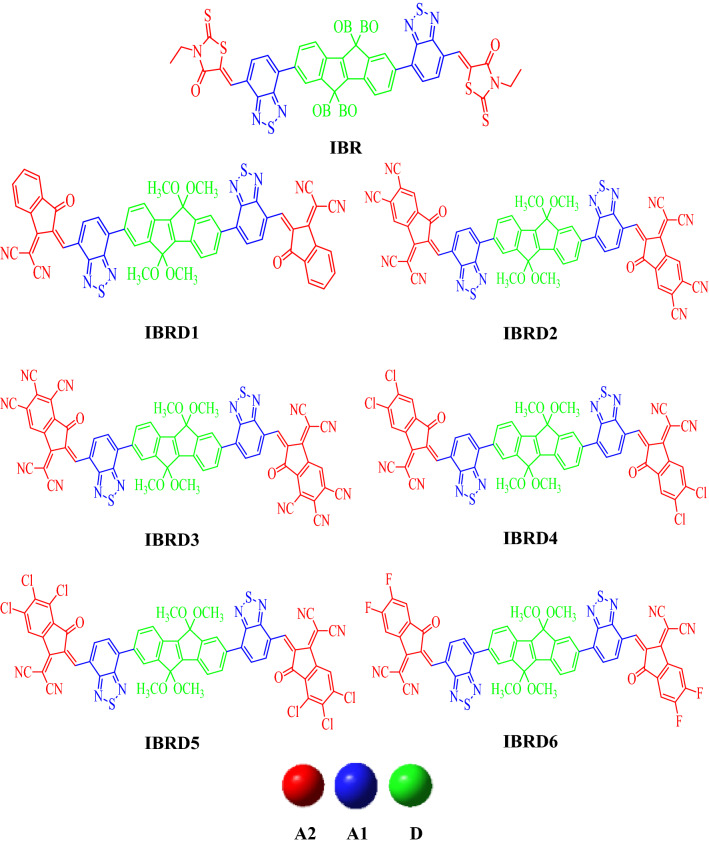
Molecular structures of **IBR** and **IBRD1**–**IBRD6** molecules.

In current investigation, **IBR**, used as a reference molecule, consists on an indenoindene-core that behaves as a donor (D) unit and flanked by A1 (4 methylbenzo[c][1,2,5]thiadiazole) and A2 (3-ethyl-5-methylene-2-thioxothiazolidin-4-one). We substituted the terminal acceptor species (A2) of the **IBR** with various reported acceptors to design **IBRD1**–**IBRD6** species (Fig. 2) and inspected the acceptor units influence on optoelectronic and photophysical properties of **IBR**. The optimization with frequency analyses were performed for all the compounds and imaginary frequency was not present in any of the compounds (Tables S22–S28). The optimization with frequency analyses based graphs as well as optimized structures are presented in Figure S1. The absence of any negative frequency in all compounds confirm the accuracy of the optimized molecular structure at true minima. Moreover, their cartesian coordinates are displayed in Tables S1–S7.

### Frontier molecular orbital (FMO) investigations

FMO investigation is considered a crucial factor for detecting the photo-electronic properties of OSCs^48^. It is presumed that according to the highest occupied molecular orbital (HOMO) and lowest unoccupied molecular orbital (LUMO) distribution pattern, the charge transfer in photovoltaic OSCs varies significantly. According to valence band theory, the LUMO and HOMO are considered as conduction and valence bands, respectively. The difference of energy between HOMO/LUMO has been explained as the bandgap (*E*_*g*_)^49–53^. The proficiency of OSCs power conversion is fairly reliant on the energy bandgap as there would be a high photovoltaic response of a material with a low *E*_*g*_ and vice versa. Herein, molecular orbital energies and their *E*_*g*_ for entitled compounds are calculated as shown in Table 1.

**Table 1:** Computated energy of HOMO*/*LUMO, and *E*_*g*_ (*E*_LUMO_–*E*_HOMO_) of studied compounds.

Compound	HOMO	LUMO	*E*_*g*_
**IBR**	− 5.981	− 3.247	2.734
**IBRD1**	− 6.030	− 3.502	2.528
**IBRD2**	− 6.427	− 4.161	2.266
**IBRD3**	− 6.531	− 4.405	2.126
**IBRD4**	− 6.155	− 3.714	2.441
**IBRD5**	− 6.154	− 3.739	2.415
**IBRD6**	− 6.141	− 3.664	2.477

All out put files of entitled compounds were accomplished by Gaussian 09 version D.01 (https://gaussian.com/g09citation/).

**Figure 3:**
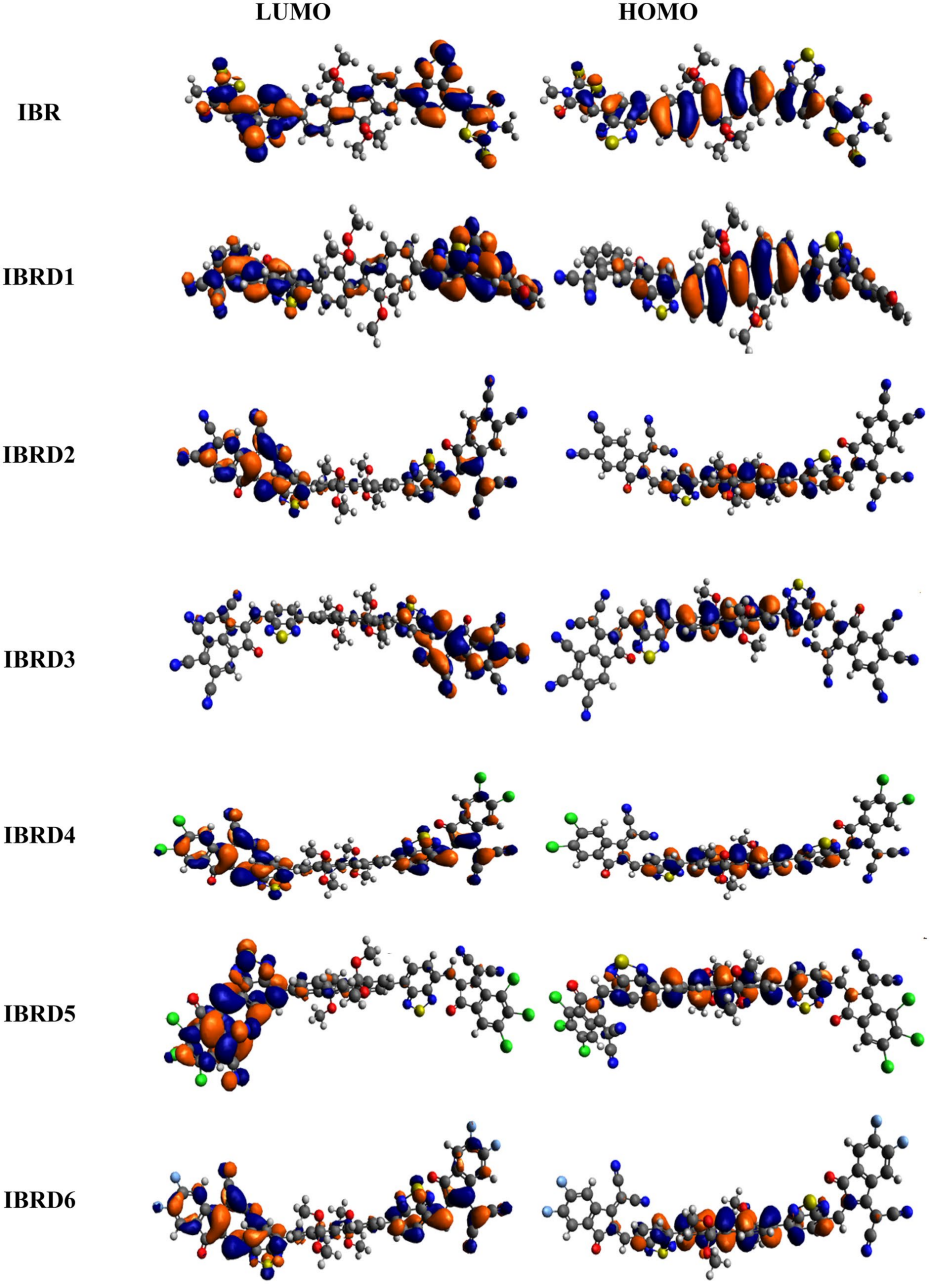
Pictorial representation of FMOs for **IBR** and **IBRD1**–**IBRD6** drawn with the help of Avogadro software, Version 1.2.0. (http://avogadro.cc/). All out put files of entitled compounds were accomplished by Gaussian 09 version D.01 (https://gaussian.com/g09citation/).

In **IBR**, 2.734 eV band gap is studied with − 5.981 and − 3.247 eV energies of HOMO and LUMO, respectively, in a closed relationship with experimental value (2.23 eV)^14,35^. Interestingly, a reduction in *E*_*g*_ has been examined in designed chromophores. The energies for HOMO are found to be − 6.030, − 6.427, − 6.531, − 6.155, − 6.154, and − 6.141 eV, respectively, for **IBRD1**–**IBRD6**, while for LUMO are − 3.502, − 4.161, − 4.405, − 3.714, − 3.739 and − 3.664 eV, respectively (Table 1). The energy gap shows a reduction when the terminal acceptor of **IBR** is modified in **IBRD1**, where the combined effect of enlargement in resonance along with electron-withdrawing effect of cyano (–CN) group stabilized the chromophore by lowering its bandgap. Furthermore, a decrease in *E*_*g*_ is examined for **IBRD2**–**IBRD3** when the number of electron-withdrawing groups (–CN) increased (Table 1). Consequently, an increase in the energy gap is also examined when the cyano group is replaced by the chloro group in **IBRD4**, as cyano is more inductive effect than chloro (–CN > Cl)^54^. The bandgap starts diminishing in **IBRD5** than **IBRD4** as the number of the electron-withdrawing groups (–Cl) increased (Fig. 2). Contrarily, a larger value of *E*_*g*_ is exhibited by **IBRD6**, 2.477 eV, as chloro group on terminal acceptor replaced with fluoro (–F) group. This might be due to the resonance effect that may compete with the inductive effect as F and Cl groups are electron donating due to the resonance effect (Cl > F)^55^. Overall, the reduction in the bandgap with terminal electron-withdrawing groups was found as F > Cl > CN. Among all the chromophores, it is inferred that **IBRD3** has a narrow energy gap as it has three cyano groups that powerfully attract the electronic cloud toward themselves and lower the band gap between orbitals. However, the decreasing *E*_*g*_ order of **IBR** and **IBRD1**–**IBRD6** is **IBR > IBRD1 > IBRD6 > IBRD4 > IBRD5 > IBRD2 > IBRD3**. Additionally, the dispersion pattern of electron density in LUMO and HOMO on the surface of both **IBR** and their fabricated molecules are shown in Fig. 3. In **IBR** the charge density is located all over the chromophore but significantly concentrated over the central indenoindene-core (donor) in HOMO while end-capped acceptor units in LUMO. Similarly, in all designed molecules charge density for HOMO exists over donor (indenoindene) unit and A1 while in LUMO on the terminal acceptors moieties. A relatively lower *E*_*g*_ between orbitals and effective CT from D to terminal A is examined in derivatives than that of reference which indicates them to be efficient materials for solar cells.

### Density of state (DOS)

**Figure 4:**
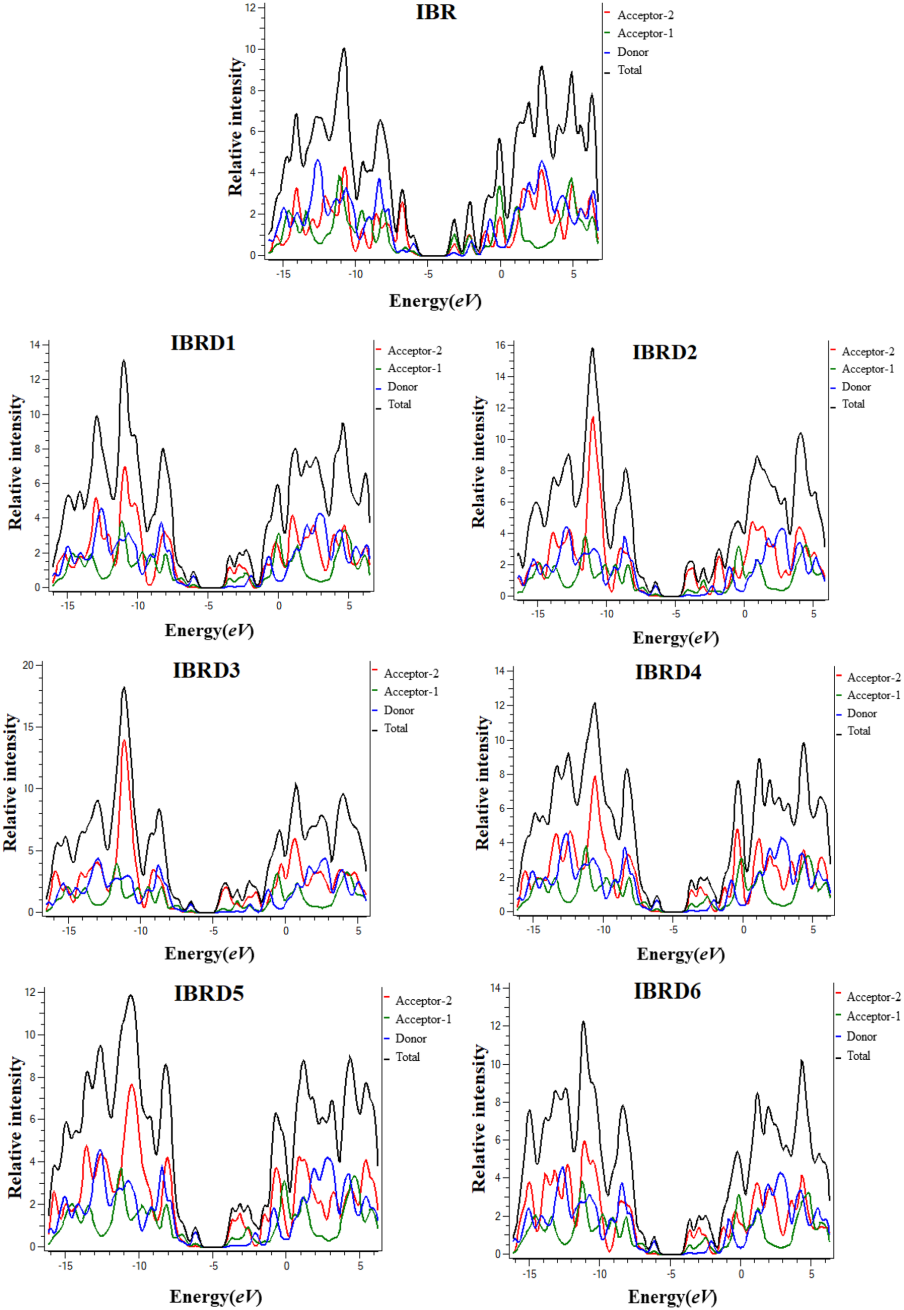
Graphical representation of the density of states (DOS) of studied chromophores drawn by utilizing PyMOlyze 1.1 version (https://sourceforge.net/projects/pymolyze/). All out put files of entitled compounds were accomplished by Gaussian 09 version D.01 (https://gaussian.com/g09citation/).

The density of state (DOS) is the number of different states that electrons will occupy at a given energy level. For energy levels, a high DOS value indicates that numerous states are vacant. The DOS zero value exhibits that there are no states available for occupation at any energy level. DOS computations allow the broad distribution of states as a function of energy to be measured and *E*_*g*_ can also be determined^56^. Thus, DOS helps in the manifestation of evidence discussed in FMOs and percentage influences about HOMO and LUMO charge densities. Herein, to investigate DOS, **IBR** and **IBRD1**–**IBRD6** are divided into three fragments, i.e., A1, donor, and A2. In DOS spectra, the scattering pattern of the donor is manifested by a blue line, whereas the green and red lines exhibit the scattering pattern of acceptor-1 and acceptor-2, respectively (Fig. 4). The positive values along the x-axis specify LUMO (conduction band), while negative values express the HOMO (valence band), and the distance between conduction and valence band is expressed as a bandgap^44,57^.

For **IBR,** the Acceptor-1 contributes 20.8% to HOMO and 57.3% to LUMO, whereas, Acceptor-2 contributes to HOMO 14.2% and 30.4% to LUMO. Similarly, Donor contributes to HOMO, 65.0%, and LUMO, 12.3% in **IBR**. The Acceptor-1 contributes 19.4%, 17.7%, 17.6%, 18.8%, 18.5%, and 18.7% to HOMO and 31.5%, 22.1%, 18.9%, 27.8%, 25.4% and 28.4% to LUMO in **IBRD1**–**IBRD6**, respectively. Similarly, acceptor-2 contributes 6.7%, 7.4%, 7.5%, 6.7%, 6.2% and 6.3% to HOMO, while 61.4%, 73.0%, 76.7%, 66.1%, 69.4% and 65.5% to LUMO for **IBRD1**–**IBRD6**, respectively. In the same way, donor contributes 74.0%, 74.9%, 74.9%, 74.5%, 75.2% and 75.0% to HOMO whereas 7.1%, 4.9%, 4.4%, 6.1, 5.2, and 6.2% to LUMO for **IBRD1**–**IBRD6**, accordingly. DOS speculates that various electron-withdrawing acceptor groups are accountable for different scattering patterns of electron densities. Further, these electronic transitions are also responsible for intramolecular charge transfer. Figure 4 shows that in all chromophores for HOMO the highest peak for charge density is observed at the donor part in the range of − 6 to − 6.5 eV while in LUMO, it appears in A2 units at − 4 eV. Hence, these energy ranges are significant and demonstrated that donor and terminal acceptor moieties are mainly responsible to arise HOMO and LUMO, respectively in designed chromophores which also supported the FMO investigation.

### UV–visible absorption spectra

The UV–visible absorption properties were determined by utilizing M06/6-311G(*d*,*p*) level in chloroform and gaseous phase to elucidate the optical properties of **IBR** and **IBRD1**–**IBRD6** (Tables 2, 3; S8–S21). In addition, different parameters comprising oscillator strengths ($f_{os}$), transition energy, and molecular orbital transitions were investigated.

**Table 2:** Wavelength, energy, and oscillator strength of reference and designed molecules in gaseous phase.

Compounds	$\lambda$ (nm)	*E* (eV)	*f*	MO contributions
**IBR**	569	2.178	1.684	H → L (94%)
**IBRD1**	617	2.007	1.410	H → L (93%)
**IBRD2**	690	1.794	1.267	H → L (94%)
**IBRD3**	734	1.689	1.145	H → L (93%)
**IBRD4**	641	1.933	1.364	H → L (93%)
**IBRD5**	645	1.921	1.132	H → L (91%)
**IBRD6**	630	1.968	1.295	H → L (93%)

MO = molecular orbital, HOMO = H, LUMO = L, ***f* = **oscillator strength.

**Table 3:** Wavelength, energy and oscillator strength of reference and designed compounds in solvent (chloroform).

Compounds	$\lambda$ (nm)	*E* (eV)	*f*	MO contributions
**IBR**	575	2.154	1.977	H → L (92%)
**IBRD1**	619	2.001	1.627	H → L (91%)
**IBRD2**	694	1.786	1.457	H → L (91%)
**IBRD3**	745	1.663	1.299	H → L (92%)
**IBRD4**	643	1.928	1.572	H → L (91%)
**IBRD5**	646	1.918	1.282	H → L (89%)
**IBRD6**	628	1.974	1.533	H → L (90%)

MO = molecular orbital, HOMO = H, LUMO = L, *f* = oscillator strength, values in parenthesis are experimental.

**Figure 5:**
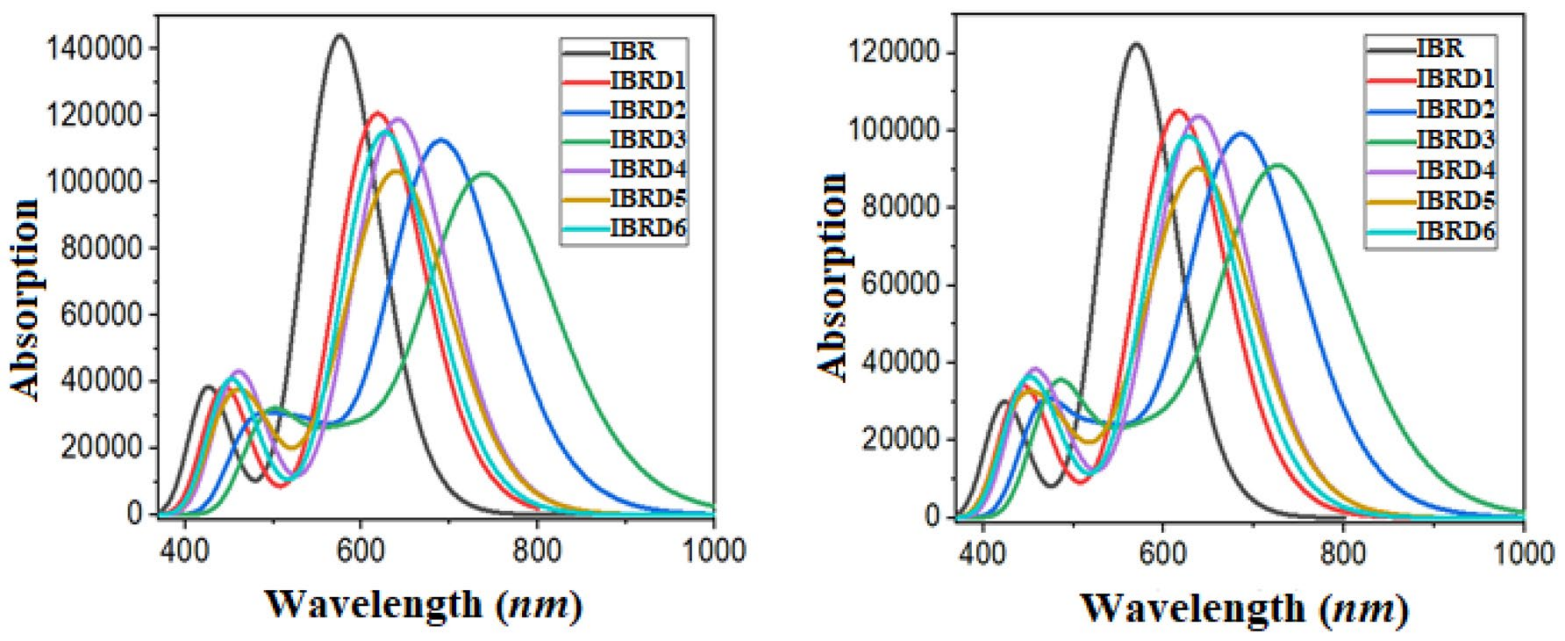
Absorption spectra of **IBR** and **IBRD1**–**IBRD6** in the gaseous phase (left) and chloroform solvent (right) made by using origin 8.5 version (https://www.originlab.com/). All out put files of entitled compounds were accomplished by Gaussian 09 version D.01 (https://gaussian.com/g09citation/).

Donor–acceptor systems with low energy offset and high photoluminescence show improved performance for high-open-circuit-voltage OSCs^58,59^. Our results exhibit that efficient electron-withdrawing terminal units with a prolonged conjugation lower the bandgap and allowed the IBRD1–IBRD6 molecules to exhibit smaller excitation energies than IBR, with greater absorption spectra in the visible region (Fig. 5). Among all the derivatives of IBR, the lower value of λmax is examined in IBRD1 which then increased in IBRD6 as the introduction of the fluoro group with cyano group on the terminal acceptor unit.These groups enhance the electron-withdrawing effect in IBRD6, which reduced the energy gap between orbitals and hence, lowers excitation energy with a broader absorption band is examined. Further, a larger absorption band is found in IBRD4–IBRD5, where the fluoro groups are replaced with chloro groups. Higher red shift is examined in IBRD2–IBRD3 when chloro groups are replaced with a more electron-withdrawing cyano group which diminished the E_g_. The same trend for absorption is examined in chloroform for all entitled chromophores, but interestingly, in solvent larger bathochromic shift is investigated, which may be due to the polarity of the solvent. Among all derivatives, IBRD3 shows the maximum absorption due to the presence of powerful electron-withdrawing five cyano groups on the terminal acceptor. The increasing absorption pattern is in order of IBR < IBRD1 < IBRD6 < IBRD4 < IBRD5 < IBRD2 < IBRD3 which is inversely related with E_g_. The generated absorption spectra of IBR and IBRD1–IBR D6 in gaseous chloroform are shown in Fig. 5. The results show that all designed compounds exhibit better optical properties than IBR. It is therefore evident that structural modeling of the parent molecule with strong acceptor units, chromophores with reduced bandgap and broader absorption spectra can lead to the development of appealing OSCs materials.

### Reorganization energy (RE)

Measuring the reorganization energy (RE) of the compounds is one of the simplest ways to test CT (charge transfer) properties^60^. The RE factor defines the position of electron mobility and holes as it directly correlates with the mobility of charges. Therefore, if a compound has low RE, it has elevated mobility of electrons and holes or vice versa. By adjusting parameters, fluctuations in reorganization power occur, but these fluctuations are highly dependent on types of two phases such as number of anions and cations. Cationic structure accords with the hole mobility, while anionic structure accords with the movement of electrons from particular ends. RE is partitioned into two phases; one arrangement inside RE and the other with outer RE. Internal reorganizational energy (*λ*_*int*_.) is connected with the inner climate of particles and outside reorganizational energy (*λ*_*ext*_.) should be identified with the outside environment of an atom. As outside climate effect is less relevant in this context so we are excluding outer RE for this manuscript. Charge transfer and reorganization energy have an inverse relation, so if the reorganization energy is low, the system initiates a significant amount of charge transfer^61–65^. Therefore, reorganization energies $\lambda_{{\text{e}}}$ and $\lambda_{h}$ ($\lambda_{{\text{e}}} =$ RE of electron) and ($\lambda_{{\text{h}}} =$ RE of hole) are calculated for entitled chromophores with the help of following equations:

1 $$ \lambda_{e} = \left[ {{\text{E}}_{0}^{ - } - {\text{E}}_{ - } } \right] + \left[ {{\text{E}}_{ - }^{0} - {\text{E}}_{0} } \right] $$

2 $$ \lambda_{h} = \left[ {{\text{E}}_{0}^{ + } - {\text{E}}_{ + } } \right] + \left[ {{\text{E}}_{ + }^{0} - {\text{E}}_{0} } \right] $$

Here, $E_{0}^{ + }$ and $E_{0}^{ - }$ are RE of the cation and anion, respectively, computed at the optimized state of a neutral compound. $E_{ + }$ and $E_{ - }$ are the RE of cation and anion, respectively.

The *λ*_*e*_ for **IBR** is calculated to be 0.00882 eV and all derivatives have the higher value of *λ*_*e*_ except **IBRD2** and **IBRD3** as shown in Table 4. According to literature, the compounds with higher value of *λ*_*e*_ exhibited lower rate of charge transfer^14,56,57,66^. Therefore, the results from Table 4 indicated that higher rate of electron mobility is presented between donor and acceptor units in **IBRD2** and **IBRD3** among all the studied compounds as they have the lower value of *λ*_*e*_. While **IBRD1**, **IBRD5** and **IBRD6** have the higher value of electron reorganization energies than reference so expressed lower electron mobility rate than the parent molecule. **IBRD4** and reference compound have almost equal electron charge transfer rate as they have comparable values of *λ*_*e*_ 0.009976 and 0.00882 eV, respectively. Overall decreasing order of *λ*_*e*_ is **IBRD3** > **IBRD2** > **IBR**. > **IBRD4** > **IBRD5** > **IBRD1** > **IBRD6**. Similarly, *λ*_*h*_ calculated for reference is lower than all its derivatives which indicates that among all the designed compounds higher rate for hole transportation is present than parent chromophore (see Table 4). The higher value of *λ*_*h*_ in designed compounds might be due to their higher ionization potential which inhibits the movement of the holes. Overall, a higher value of *λ*_*h*_ and lower *λ*_*e*_ is found in all designed **IBRD1**–**IBRD6** molecules, this making them excellent candidates for electron mobility and appealing acceptors for OSCs. Previosuly, heterojunction interface models with nonfullerene acceptors show better light harvesting capability and intramolecular charge transfer properties along with lower burn-in degradation^67,68^.

**Table 4:** Reorganization energies ($\lambda_{e}$ and $\lambda_{h}$) of IBR and IBRD1–IBRD6.

Compounds	$\lambda_{{\text{e}}}$ (eV)	$\lambda_{h}$ (eV)
**IBR**	0.00882	0.009662
**IBRD1**	0.010651	0.011942
**IBRD2**	0.00744	0.012576
**IBRD3**	0.005421	0.011913
**IBRD4**	0.009976	0.012235
**IBRD5**	0.010489	0.011968
**IBRD6**	0.010971	0.012327

All out put files of entitled compounds were accomplished by Gaussian 09 version D.01 (https://gaussian.com/g09citation/).

### Exciton binding energies and transition density matrix analysis

**Figure 6:**
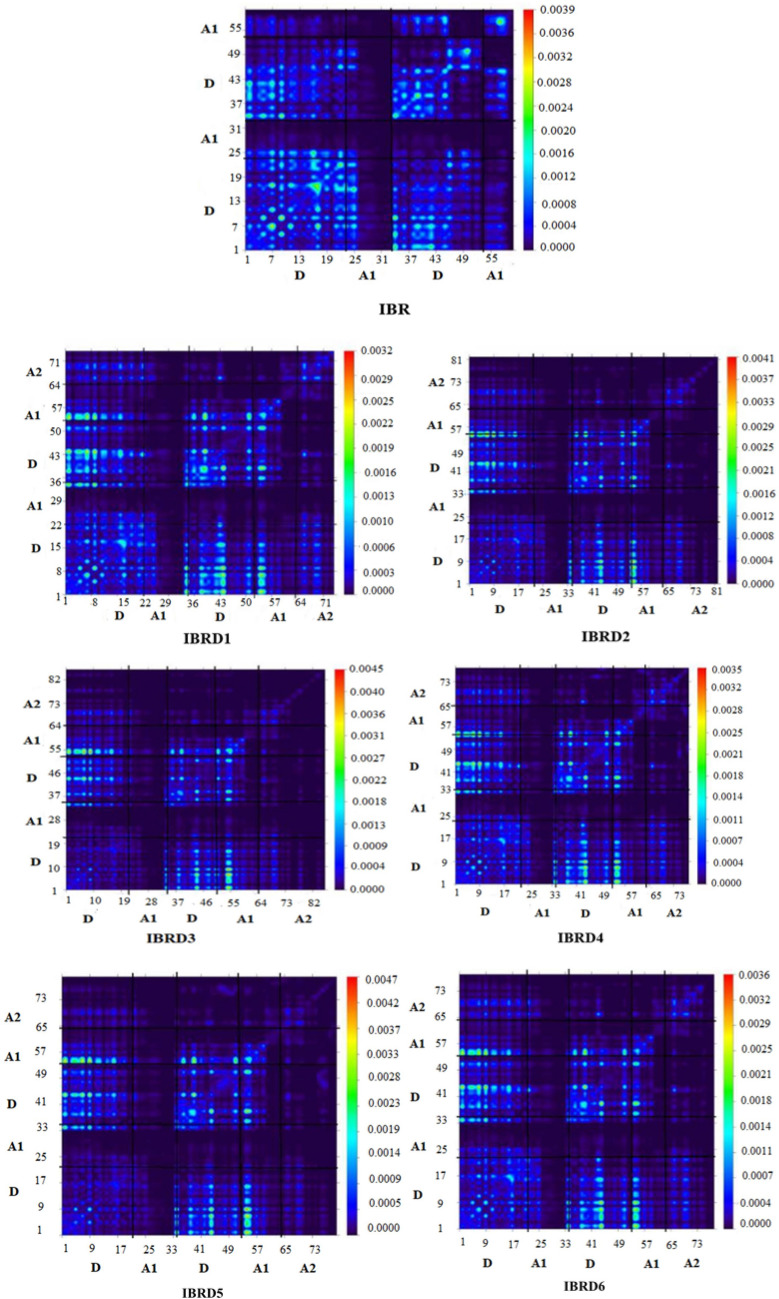
TDM of the **IBR** and **IBD1**–**IBRD6** at the S1 state drawn with the help of Multiwfn 3.7software (http://sobereva.com/multiwfn/). All out put files of designed compounds were accomplished by Gaussian 09 version D.01 (https://gaussian.com/g09citation/).

The tool for measuring and evaluating the transmission of the charge of electrons in an excited state is known as transition density matrix (TDM). In an excited state, it supports to explain donor–acceptor unit interactions, electronic excitation, hole-electron localization and delocalization^56,69^. As a consequence of extremely limited contribution, hydrogen atoms are excluded during calculation. The nature of the transition is shown in TDM diagrams for all investigated molecules in Fig. 6. To calculate the TDM, we made fragments of designed molecules like Donor (central core: **D**) and Acceptors (end-capped groups: **A1, A2**) units.

In the scattered form, TDM diagrams shows the presence of charge movement. By considering the FMO and DOS analysis, transfer of charge occurs significantly all over the molecule in designed chromophores. This CT brings considerable changes in TDM heat maps. TDM plots exploited that excitations are significantly confined on D (indenoindene) units and then these excitations diagonally extend via A1 and A2 as though electron–hole pair started to build along diagonally without trapping in all investigated molecules. Binding energy (*E*_*b*_) is another favorable factor that assists in determining the capacity for exciton dissociation, photo-electronic properties, and efficiency of OSCs. The PCE of OSCs and the parting rate of charges depend on the binding energies (*E*_*b*_). Further, *E*_*b*_ is also linked to energy driving force (∆E). ∆E is the difference of LUMOs of acceptor and donor and it should be greater than 0.3 eV, for effective exciton split and charge transfer at DA interface^69,70^. The band gap difference of the optical and electrical energies gives the exciton binding energies. By using Eq. (3) we can calculate *E*_*b*_^71^_*.*_

3 $$ E_{b} = E_{H - L} - E_{opt} $$

In Eq. (3) *E*_*H–L*_ = the *E*_*g*_ of HOMO/LUMO. *E*_*opt*_ = minimum amount of energy required for the first excitation, attained from S_0_ to S_1_.

It is a significant instrument that tests the columbic forces, the interaction between *e* (electron) and *h* (hole). There is a direct relationship between *E*_*b*_ and coulombic hole-electron interaction, which has an inverse relation with exciton dissociation in the excited state^72^. A molecule with low *E*_*b*_ indicates low columbic contact between *h* and *e* that triggers high dissociation of arousal in an excited state. The **IBR** has a higher value of exciton *E*_*b*_ while **IBRD3** expressed the lowest value of exciton *E*_*b*_ 0.437 eV along with smaller *λ*_*e*_, compared to the reference and other designed compounds indicating the presence of the greater amount of charges, leading to a higher degree of charge separation in the S1 state observed in Table 5. The decreasing *E*_*b*_ order is obtained to be **IBR > IBRD1 > IBRD6 > IBRD4 > IBRD5 > IBRD2 > IBRD3** (Table 5). Because of the low value of *E*_*b*_, all the derivatives expressed a higher degree of charge partition, hence can act as appealing OSCs material.

**Table 5:** Calculated *E*_*H–L*_*, E*_*opt*_*,* and *E*_*b*_ of reference and designed molecules.

Compounds	*E*_*H–L*_ (eV)	*E*_*opt*_ (eV)	*E*_*b*_ (eV)
**IBR**	2.734	2.178	0.556
**IBRD1**	2.528	2.007	0.521
**IBRD2**	2.266	1.794	0.472
**IBRD3**	2.126	1.689	0.437
**IBRD4**	2.441	1.933	0.508
**IBRD5**	2.415	1.921	0.494
**IBRD6**	2.477	1.968	0.509

All out put files of entitled compounds were accomplished by Gaussian 09 version D.01 (https://gaussian.com/g09citation/).

### Open circuit voltage (*V*_oc_)

The term *V*_*oc*_ has its significance in organic solar cells^73^ as OSCs working capability and performance are estimated by examining its *V*_*oc*_. It can be defined as the total quantity of current that can be passed through any optical device^74^. The *V*_*oc*_ is maximum voltage substantially at zero-current levels. Recombination in devices can be achieved with the help of saturation current and light generated current; ultimately, *V*_*oc*_ depends on these two factors. Open circuit voltage has an inverse relation with the *E*_*g*_ of donor and acceptor compounds, respectively^75,76^. A higher value of *V*_*oc*_ can be attained if the LUMO level of the acceptor has a higher energy value and the HOMO of the donor have a lower value^77^. Charber and his coworkers^78,79^ proposed an equation to calculate the *V*_*oc*_ values, the open circuit values of all investigated compounds are calculated by Eq. (4).

4 $$ {\text{V}}_{{{\text{oc}}}} = \left( {\left| {{\text{E}}_{{{\text{HOMO}}}}^{{\text{D}}} } \right| - \left| {{\text{E}}_{{{\text{LUMO}}}}^{{\text{A}}} } \right|} \right) - 0.3 $$

where *E* is the energy and 0.3 is a constant observed from simplifying voltage drop factors^60,80^ The main idea of *V*_*oc*_ is to align the LUMO of designed molecules, including **IBR**, with the HOMO of a well-acknowledged **PBDBT** donor. The results obtained are tabulated in Table 6.

**Table 6:** Computed *V*_*oc*_ values and energy gap values of **IBR** and **IBRD1**–**IBRD6.**

Compounds	**IBR**	**IBRD1**	**IBRD2**	**IBRD3**	**IBRD4**	**IBRD5**	**IBRD6**
***V***_***oc***_	1.854	1.599	0.94	0.696	1.387	1.362	1.437
***∆E***	2.154	1.899	1.24	0.996	1.687	1.662	1.95

∆E = band gap between the orbitals (HOMO/LUMO) of donor/acceptor complexes. All out put files of entitled compounds were accomplished by Gaussian 09 version D.01 (https://gaussian.com/g09citation/).

**Figure 7:**
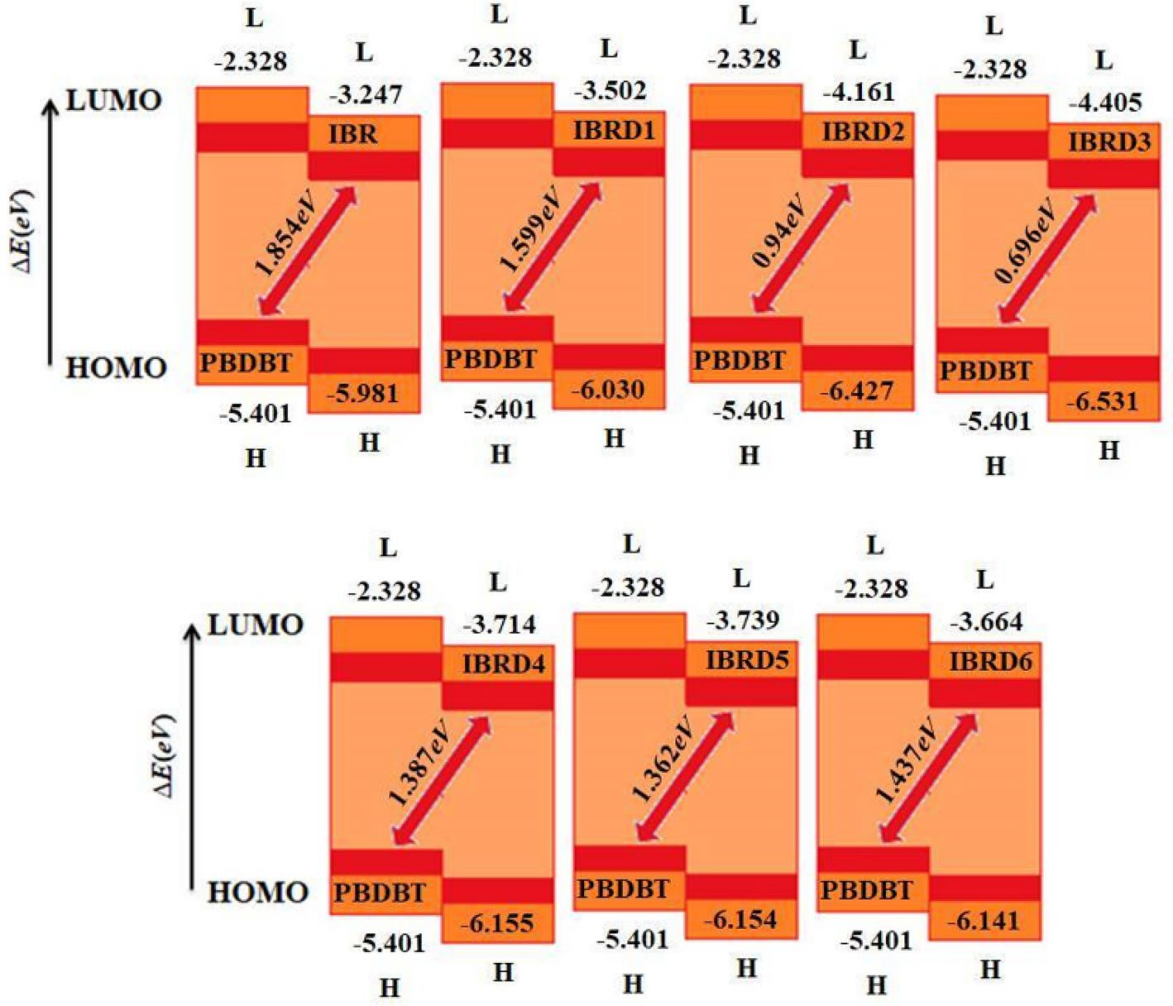
The open-circuit voltage (*V*_*oc*_) of **IBR** and **IBRD1**–**IBRD6** with respect to the donor **PBDBT.** All out put files of entitled compounds were accomplished by Gaussian 09 version D.01 (https://gaussian.com/g09citation/).

The band gap between the orbitals (HOMO/LUMO) of donor/acceptor complexes is found to be 2.154, 1.899, 1.24, 0.996, 1.687, 1.662, and 1.95* eV*, respectively, for **IBR** and **IBRD1**–**IBRD6** (Table 6; Fig. 7). This shows that **PBDBT:IBR** complex has the highest energy gap value than all other derivative complexes. The *V*_*oc*_ of **IBR** with respect to HOMO_PBDBT_‒LUMO_Acceptor_ is 1.854 V. *V*_*oc*_ of **IBRD1**–**IBRD6** are 1.599, 0.94, 0.696, 1.387, 1.362 and 1.437 V, respectively. All the designed compounds expressed comparable *V*_*oc*_ value with respect to reference molecules. The decreasing order of *V*_*oc*_ values is: **IBR** > **IBRD1** > **IBRD6** > **IBRD4** > **IBRD5** > **IBRD2** > **IBRD3**. An acceptor species with lower lying LUMO causes greater *V*_*oc*_. A low lying LUMO orbital means that the electron can easily be transferred between the donors to the acceptor unit. In addition, the energy gap between the HOMO and LUMO is also important for the transition of electrons between the donors to the acceptor unit and enhances the PCE. It is clear that in all designed molecules, **IBRD1** has higher *V*_*oc*_ than other designed molecules, thus possessing better optoelectronic properties. The higher *V*_*oc*_ of **IBRD1** is due to the higher LUMO and lower HOMO values. The above discussion concludes that all designed acceptor molecules are suitable candidates for use in OSCs due to better optoelectronic properties when aligned with the HOMO_PBDBT._

### Charge transfer analysis

**Figure 8:**
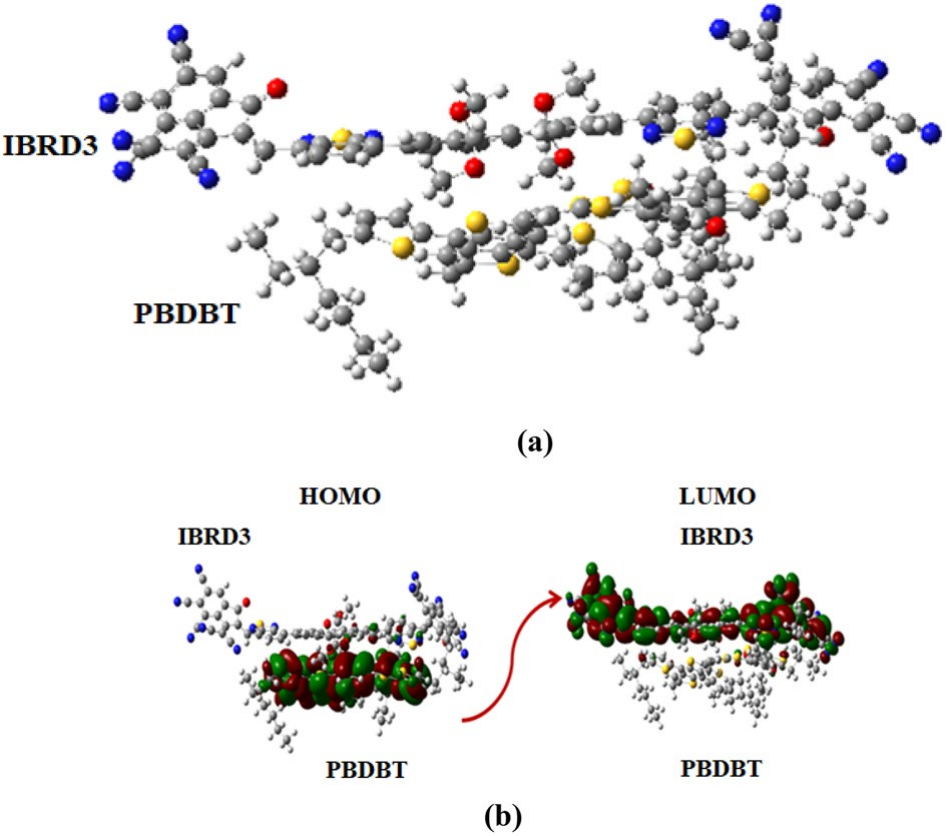
Optimized geometry of **PBDBT:IBRD3** charge transfer between (**a**) ${\text{HOMO}}_{{{\text{PBDBT}}}}$ and (**b**) ${\text{LUMO}}_{{{\text{IBRD}}3}}$. at M06/3-21G are made with the help of GaussView 5.0 and Gaussian 09 version D.01 (https://gaussian.com/g09citation/).

To understand the phenomena of charge transfer between our designed acceptor chromophores, we utilized a well-known donor **PBDBT** polymer. For this purpose, we blend the **IBRD3** molecule with **PBDBT** polymer because of its lowest transition energy, highest *λ*_*max*_, good electron and hole mobility values among **IBRD1**–**IBRD6**. Additionally, **IBRD6** is also utilized to make a complex with **PBDBT** polymer as it exhibited a higher value of *V*_*oc*_ among all derivatives (Figure S2a, b). The optimization of both complexes (i) **PBDBT:IBRD3** and (ii) **PBDBT:IBRD6** is implemented at M06 and $\omega$ B97XD functionals with 3-21G basis set and structures are shown in Figs. 8a and S2(a) for the best transfer of charge; we put our designed chromophores parallel to the donor polymer (Fig. 8b and S2). It is clear from Fig. 8b that the charge density for HOMO is located over the donor **PBDBT** polymer while LUMO is located over the **IBRD3,** which indicates that an excellent transfer of electronic cloud from donor **PBDBT** polymer towards **IBRD3** chromophore. The same phenomena are observed for **PBDBT:IBRD6** (Figure S2b). This excellent charge transfer revealed that our designed chromophores are suitable non-fullerene solar cell acceptors, which may play a significant role in designing optoelectronic devices.

## Conclusion

A series of indenoindene based A2-A1-D-A1-A2 architecture of novel NF-SMAs (**IBRD1**–**IBRD6)** was designed from organic chromophore **IBR**. These **IBRD1**–**IBRD6** chromophores were obtained by the structural modification of terminal acceptor units. The effect of various A units was examined on photovoltaic properties and a comparable relation is found between parent chromophore and their derivatives. Interestingly, all the derivatives showed a broader spectrum with a smaller bandgap than the parent molecule. FMO, DOS and TDM findings reveals that an effective charge is transfer from donor moiety to acceptor units. Further lower value of *λ*_*e,*_ indicated higher rate of electron mobility in all designed chromophores.The *V*_*oc*_ calculated with respect to **PBDBT** for **IBRD1**–**IBRD6** was 1.854, 1.599, 0.94, 0.696, 1.387, 1.362, and 1.437 V, respectively. In all newly designed molecules, **IBRD3** exhibits the highest optical absorption wavelength (754 nm) with the lowest energy band gap (2.126 eV). All molecules with lower binding energies make the higher exciton dissociation in an excited state, which eventually causes a high transfer rate of charge. These photovoltaic properties suggest that all the newly designed molecules were excellent acceptor candidates for obtaining high PCE in organic solar cells.

## Supplementary Information

Supplementary Information.
