# Supplementary material for: Molecular engineering of indenoindene-3-ethylrodanine acceptors with A2-A1-D-A1-A2 architecture for promising fullerene-free organic solar cells
Source: Sci Rep. 2021 Oct 13;11:20320. doi: 10.1038/s41598-021-99308-7 (PMC8514561; doi:10.1038/s41598-021-99308-7)
Supplement: Supplementary file 1 — Supplementary Information. [file 41598_2021_99308_MOESM1_ESM.docx]

**Molecular Engineering of Indenoindene-3-ethylrodanine Acceptors with A2-A1-D-A1-A2 Architecture for Promising Fullerene-Free Organic Solar Cells**

Muhammad Khalid,*^1^ Momina, ^1^ Muhammad Imran,^2^ Muhammad Fayyaz ur Rehman^3^, Ataualpa Albert Carmo Braga^4^, Muhammad Safwan Akram*^5^

*^1^Department of Chemistry, Khawaja Fareed University of Engineering & Information Technology, Rahim Yar Khan, 64200, Pakistan*

*^2^Department of Chemistry, Faculty of Science, King Khalid University, P.O. Box 9004, Abha 61413, Saudi Arabia.*

*^3^Institute of Chemistry, University of Sargodha, Sargodha, 40100, Pakistan*

*^4^Departamento de Química Fundamental, Instituto de Química, Universidade de São Paulo, Av. Prof. LineuPrestes 748, São Paulo, 05508-000, Bra`zil*

*^5^School of Health and Life Sciences, Teesside University, Middlesbrough, TS1 3BA, UK and National Horizons Centre, Teesside University, Darlington, DL1 1HG, UK*

*Corresponding authors E-mail addresses:

Dr. Muhammad Khalid ([muhammad.khalid@kfueit.edu.pk](mailto:muhammad.khalid@kfueit.edu.pk); [Khalid@iq.usp.br](mailto:Khalid@iq.usp.br))

Dr. Muhammad Safwan Akram ([Safwan.akram@tees.ac.uk](mailto:Safwan.akram@tees.ac.uk))

**Cartesian Co-ordinates**
Table S1: Cartesian Co-ordinates of reference molecule (IBR)

**Atoms X Y Z**

C 3.57055100 -2.23398200 -0.40183900

C 4.29152000 -1.03877100 -0.34850200

C 3.58120100 0.17762100 -0.34058700

C 2.21167000 0.16627400 -0.31928000

C 1.49435300 -1.04245400 -0.36262200

C 2.18166900 -2.24476000 -0.41914600

H 4.10866800 -3.17362100 -0.44011700

H 4.11969400 1.11829700 -0.40779400

H 1.65081400 -3.18735800 -0.49777300

C 0.08273500 -0.70012000 -0.41352100

C 1.24959500 1.35039200 -0.38231700

C -0.08439300 0.63288800 -0.46782200

C -1.25272900 -1.41676500 -0.49255800

C -1.49593000 0.97644100 -0.50547100

C -2.21541100 -0.23108200 -0.52752400

C -3.58483800 -0.23973000 -0.46909200

H -4.12553300 -1.18115800 -0.43849300

C -2.17952900 2.18162800 -0.46489200

H -1.64590200 3.12415000 -0.40779600

C -3.56806200 2.17291300 -0.45866600

H -4.10428100 3.11433900 -0.43207000

C -4.29095300 0.97786500 -0.44710000

C -5.75418800 0.96976300 -0.34578900

C -6.58227900 1.94463700 -0.99104600

C -6.42206200 0.01422300 0.38665900

C -8.01731900 1.89620100 -0.86019500

C -7.82544000 -0.03233200 0.51803400

H -5.84140000 -0.72828100 0.92542800

C -8.66586800 0.88223200 -0.08033500

H -8.23458300 -0.81868600 1.14311300

N -6.16475000 2.94317300 -1.76639000

N -8.64148700 2.85760000 -1.53037100

S -7.49007400 3.74018400 -2.26498200

O 1.17206300 2.13913700 0.77433700

O 1.64239700 2.08790500 -1.50115300

O -1.63341000 -2.16635600 0.62411000

O -1.18695300 -2.19340200 -1.65629800

C 2.37014000 2.75087800 1.19589400

H 2.09868700 3.46299000 1.97644800

H 3.07153500 2.01753200 1.61594700

H 2.86167000 3.28908900 0.37537100

C 0.79643200 3.15141300 -1.87753300

H 1.31660000 3.69894400 -2.66463100

H -0.15871100 2.78601500 -2.27564200

H 0.60331600 3.83130700 -1.03803500

C -0.78956000 -3.24034400 0.97421400

H 0.16830200 -2.88621100 1.37596600

H -1.30785900 -3.80307000 1.75191700

H -0.60215700 -3.90269400 0.11969000

C -2.39248400 -2.79224300 -2.07815000

H -3.09208000 -2.04890200 -2.48030500

H -2.13033900 -3.49390600 -2.87115800

H -2.87968200 -3.34105100 -1.26198900

C 5.75521300 -1.01914300 -0.27345500

C 6.58897100 -1.98935000 -0.91982500

C 6.42182300 -0.04595000 0.43795300

C 8.02532300 -1.91495200 -0.81384100

C 7.82568400 0.02666100 0.54411600

H 5.83838100 0.69381400 0.97818000

C 8.67078400 -0.88031100 -0.05898000

H 8.23211600 0.82754700 1.15221300

N 6.17697700 -3.00575700 -1.67435400

N 8.65472500 -2.87453000 -1.48163100

S 7.50698700 -3.78644800 -2.18516200

C -10.10030700 0.91260700 0.01850900

H -10.58784700 1.75891500 -0.46555900

C 10.10708300 -0.88308600 0.01246200

H 10.60119100 -1.72894000 -0.46561800

C 10.95200400 0.00237800 0.57256400

C -10.95038500 0.05462700 0.61225400

C -12.40414400 0.32275600 0.57690000

C -12.35386200 -1.72004900 1.77589500

S -10.62916800 -1.44299800 1.47101600

S -12.90639000 -3.02589300 2.58981100

N -13.09284500 -0.70524400 1.24087000

O -12.94789900 1.26585200 0.06666400

C -14.53932000 -0.67438100 1.34414500

H -14.87778300 0.22983100 0.83857400

H -14.96881800 -1.56011800 0.86998200

H -14.84036100 -0.65967200 2.39422700

C 12.40973000 -0.23807100 0.51218400

O 12.96144100 -1.18102900 0.01033400

C 12.34386600 1.82786400 1.67001900

S 12.88686000 3.16065200 2.44589500

S 10.61905600 1.51071300 1.40779000

N 13.09154800 0.81697600 1.13986700

C 14.54020200 0.81603100 1.21345400

H 14.94248800 1.69946300 0.71200600

H 14.86278400 0.82943000 2.25715000

H 14.88556000 -0.09225500 0.71996900

Table S2: Cartesian Co-ordinates of **IBRD1**

**Atoms X Y Z**

C 3.90808900 1.95336300 -1.05045900

C 4.54952700 1.07645700 -0.17155100

C 3.76662000 0.19660100 0.60092100

C 2.40431300 0.19021600 0.45153900

C 1.76868000 1.08106800 -0.43100500

C 2.52628800 1.96637100 -1.18214200

H 4.50571900 2.64922000 -1.62723700

H 4.23689200 -0.42263900 1.35926200

H 2.05253600 2.68455400 -1.84308500

C 0.33738500 0.90279000 -0.26299300

C 1.36022400 -0.58433300 1.25537600

C 0.07566200 0.01010200 0.70735200

C -0.94265600 1.51441600 -0.79888200

C -1.35730700 -0.14429800 0.89287400

C -1.98767500 0.76591900 0.02559000

C -3.35242300 0.81679000 -0.07550500

H -3.82212400 1.45637200 -0.81651700

C -2.12644200 -1.00492000 1.66088800

H -1.66088500 -1.74458200 2.30367200

C -3.51131800 -0.92910800 1.58361900

H -4.11670100 -1.59686900 2.18471900

C -4.14635400 -0.01556500 0.73806400

C -5.60480500 0.12610300 0.69775600

C -6.51148400 -0.96616100 0.90410300

C -6.18938700 1.35543300 0.48182000

C -7.93940300 -0.77481700 0.81931500

C -7.58264600 1.54898900 0.43075900

H -5.54932900 2.22655800 0.37895600

C -8.48637700 0.51497800 0.53351300

H -7.96137700 2.55663300 0.27948500

N -6.17373900 -2.22045200 1.19483900

N -8.63600700 -1.87066800 1.08965800

S -7.55809400 -3.05023000 1.37505300

O 1.27478300 -1.95840700 0.99589700

O 1.65426800 -0.31698100 2.59494100

O -1.25757400 1.24548200 -2.13217600

O -0.82736000 2.88872300 -0.54787200

C 2.44596500 -2.70929400 1.22330000

H 2.16338800 -3.75986000 1.14269100

H 3.21558100 -2.49312900 0.47071900

H 2.85804000 -2.52494000 2.22400900

C 0.72821200 -0.79597500 3.54418100

H 1.18259100 -0.64915100 4.52506200

H -0.21452300 -0.23587700 3.50548900

H 0.52117500 -1.86417600 3.40221900

C -0.32419600 1.69276600 -3.09025400

H 0.61485400 1.12828900 -3.03049200

H -0.77490800 1.52195900 -4.06870900

H -0.11093900 2.76319900 -2.97569800

C -1.98206200 3.66468800 -0.77418800

H -2.74426200 3.48886700 -0.00311000

H -1.67078600 4.70912500 -0.72520000

H -2.41683300 3.46649600 -1.76242900

C 6.00836500 1.05970400 -0.03430900

C 6.80740800 2.24607100 -0.14103600

C 6.69664100 -0.10212900 0.23823500

C 8.23493700 2.19100200 0.04099400

C 8.09722400 -0.15915100 0.38510100

H 6.14034600 -1.03146900 0.31623800

C 8.90083200 0.95170200 0.28326500

H 8.55296400 -1.12191700 0.60155100

N 6.36432500 3.48127900 -0.36270100

N 8.83350800 3.37200600 -0.05610600

S 7.65932900 4.46093000 -0.34566300

C -9.90303200 0.78816600 0.45505800

H -10.19559000 1.73082600 0.91242800

C 10.32761300 0.94207300 0.47950800

H 10.73680700 1.81920000 0.98767300

C 12.40682800 -2.03360300 -0.12109500

C 13.21412100 -1.23224800 0.69573900

C 14.40488300 -1.68433300 1.23002700

C 14.79121500 -2.98648100 0.94950900

C 13.98901600 -3.80090300 0.15274400

C 12.79818200 -3.34130500 -0.39482200

C 11.25058500 -0.00489800 0.20292000

H 15.00325800 -1.02316700 1.84930400

H 15.71978100 -3.37853900 1.35182700

H 14.30076200 -4.82076800 -0.04891300

H 12.20049400 -4.00121600 -1.01110100

C 12.58354900 0.08957600 0.88090100

O 13.01999300 1.03001700 1.49419900

C 11.25064700 -1.23844100 -0.57636800

C 10.48656100 -1.51637100 -1.67721000

C 9.57802000 -0.58018800 -2.24354300

N 8.85706700 0.17222100 -2.74122300

C 10.61298300 -2.73008000 -2.40971700

N 10.69084600 -3.71708900 -3.00439200

C -12.92330100 -0.69489500 -1.03901600

C -11.91565100 -1.44766900 -1.64899300

C -12.18014600 -2.40416000 -2.60948900

C -13.50319500 -2.62499900 -2.96001300

C -14.52024000 -1.88668500 -2.35730700

C -14.25099300 -0.91896700 -1.39934800

C -10.86163700 0.02975400 -0.12697800

H -11.35999800 -2.95505000 -3.05948700

H -13.75331200 -3.37283700 -3.70565200

H -15.55110000 -2.06846700 -2.64423700

H -15.06873100 -0.36218200 -0.96047700

C -10.59256800 -1.04343900 -1.13543200

O -9.52696500 -1.43475300 -1.54017100

C -12.30992800 0.23869300 -0.07885200

C -12.99375500 1.09131100 0.74547000

C -14.41222100 1.20848100 0.73542600

N -15.56104800 1.32654000 0.74664700

C -12.36493900 1.93627400 1.70274900

N -11.88313600 2.63545800 2.48607400

Table S3: Cartesian Co-ordinates of **IBRD2**

**Atoms X Y Z**

C -3.83887200 -2.32381300 -1.39539000

C -4.51793800 -1.65426700 -0.37368600

C -3.76842300 -1.00473800 0.62678500

C -2.40049200 -0.99971900 0.55442200

C -1.72628400 -1.67669400 -0.47773300

C -2.45250400 -2.34653700 -1.45053400

H -4.40865500 -2.83978000 -2.15826800

H -4.27326700 -0.56427500 1.48146600

H -1.95416800 -2.90174500 -2.23738200

C -0.30362700 -1.55561300 -0.21169600

C -1.39550000 -0.43938800 1.55884600

C -0.08728300 -0.90513100 0.94571800

C 1.00444200 -2.01694800 -0.82819800

C 1.33571900 -0.78075500 1.20926000

C 2.00953900 -1.45552500 0.17535700

C 3.37678400 -1.44315700 0.09723400

H 3.88023800 -1.87877100 -0.76069800

C 2.06322000 -0.10729900 2.17869800

H 1.56545800 0.44728800 2.96643400

C 3.44979200 -0.12170900 2.11748100

H 4.01959700 0.40225300 2.87445400

C 4.12855500 -0.78752500 1.09284400

C 5.59019300 -0.83165900 1.03016400

C 6.42869600 0.24743600 1.46797700

C 6.24838900 -1.94330100 0.54305300

C 7.86237600 0.18247400 1.32518700

C 7.64670700 -2.02258200 0.43836400

H 5.66375600 -2.81119500 0.25366200

C 8.48511600 -0.96998800 0.75010800

H 8.08768000 -2.94326700 0.06463100

N 6.01656100 1.37936200 2.03423400

N 8.49126500 1.23699100 1.82520300

S 7.34502900 2.24500200 2.37946700

O -1.29821300 0.95683400 1.61624100

O -1.74918000 -0.99888600 2.78803300

O 1.35468700 -1.45366000 -2.05593200

O 0.91158000 -3.41373100 -0.88806800

C -2.47187400 1.65134800 1.97480400

H -2.18055900 2.68879100 2.14367700

H -3.21864300 1.62225000 1.17040300

H -2.91388200 1.24807700 2.89475000

C -0.85458200 -0.77407600 3.85527500

H -1.33862400 -1.15827700 4.75397200

H 0.09113200 -1.31079300 3.70707800

H -0.64697900 0.29500400 3.99033900

C 0.46015200 -1.67649300 -3.12401100

H -0.48575200 -1.14059900 -2.97500000

H 0.94437300 -1.28963900 -4.02139700

H 0.25378400 -2.74545800 -3.26172400

C 2.08653700 -4.10238400 -1.25323900

H 2.83660300 -4.07224700 -0.45179500

H 1.79893000 -5.14059400 -1.42399900

H 2.52374100 -3.69488800 -2.17354400

C -5.97964600 -1.59757600 -0.33736500

C -6.81191400 -2.67666400 -0.78537000

C -6.64345100 -0.49084800 0.15107100

C -8.24660500 -2.57493000 -0.72287900

C -8.04455400 -0.37871900 0.17836600

H -6.05876200 0.35645200 0.49628400

C -8.88185800 -1.38161100 -0.26059100

H -8.47856900 0.53415500 0.57813100

N -6.39903100 -3.85455500 -1.24742200

N -8.87900400 -3.66271300 -1.14465900

S -7.72699300 -4.72961400 -1.57194700

C 9.90285700 -1.11671300 0.56703600

H 10.26418000 -2.13113800 0.72283300

C -10.31373200 -1.31778100 -0.20916800

H -10.81900000 -2.26710800 -0.01355400

C -12.18281500 1.85992400 -0.17638900

C -13.10745100 0.93478300 0.31748600

C -14.32401800 1.30764600 0.84424400

C -14.62734700 2.66516900 0.90477200

C -13.69600100 3.61257600 0.43085000

C -12.47684300 3.21632400 -0.11864200

C -11.15846400 -0.25988800 -0.29510000

H -15.02182300 0.55927400 1.20661700

H -11.79492300 3.97881200 -0.47548400

C -12.55423000 -0.43596200 0.20328800

O -13.10612800 -1.46208900 0.50336700

C -11.01676300 1.12723100 -0.71486100

C -10.13363700 1.63479800 -1.62838300

C -9.20490700 0.82088500 -2.33372200

N -8.46133000 0.17132900 -2.93226700

C -10.13657800 3.00384600 -2.01541400

N -10.11960200 4.11706500 -2.32166200

C 12.79597400 0.88230000 -0.53557600

C 11.73275000 1.70435200 -0.91181200

C 11.90608500 2.88403500 -1.60074500

C 13.20227000 3.27351300 -1.92355600

C 14.29037400 2.45678500 -1.55084300

C 14.09429400 1.26181500 -0.85958100

C 10.80328200 -0.16569900 0.20022300

H 11.04906800 3.48819100 -1.88099000

H 14.96237400 0.66792300 -0.60267700

C 10.44454000 1.10331600 -0.49315400

O 9.35582500 1.52967500 -0.78101000

C 12.25741700 -0.29645600 0.17337900

C 13.01429300 -1.27740500 0.75286700

C 14.43557800 -1.29757400 0.68830700

N 15.58890100 -1.33189000 0.64315100

C 12.45941900 -2.36302900 1.48754100

N 12.03418100 -3.25490700 2.08556400

C -13.99474900 5.00607200 0.51365500

N -14.22420000 6.13391600 0.58064500

C -15.87384300 3.08777700 1.45432800

N -16.88616000 3.40880600 1.90338300

C 13.41963800 4.49361400 -2.62987100

N 13.57293300 5.48349900 -3.20108900

C 15.62142400 2.84952900 -1.88542400

N 16.69945200 3.15961400 -2.15164500

Table S4: Cartesian Co-ordinates of **IBRD3**

**Atoms X Y Z**

C -3.87804000 -2.68237400 -1.04377800

C -4.54462800 -1.78591000 -0.20186200

C -3.78069000 -0.93526900 0.62315600

C -2.41450500 -0.96218100 0.54554600

C -1.75334300 -1.86037200 -0.31229900

C -2.49225700 -2.72975000 -1.10092900

H -4.46006600 -3.35798000 -1.65914600

H -4.27201900 -0.30521000 1.35845200

H -2.00170000 -3.45495100 -1.74157700

C -0.32851700 -1.69109400 -0.09317700

C -1.39739500 -0.20054800 1.39336300

C -0.09878800 -0.79898500 0.88692700

C 0.97158600 -2.28406200 -0.60347200

C 1.32609100 -0.62320400 1.10152400

C 1.98836500 -1.51698100 0.24083600

C 3.35492500 -1.52995000 0.15196000

H 3.84872500 -2.15511800 -0.58609900

C 2.06220000 0.25389500 1.88364800

H 1.56948200 0.97638600 2.52583400

C 3.44814100 0.22198000 1.81354600

H 4.02833200 0.90874000 2.41809600

C 4.11626100 -0.66710800 0.96584400

C 5.57773900 -0.73406500 0.90999100

C 6.42377700 0.41018100 1.09611400

C 6.22938700 -1.92897800 0.68455200

C 7.85736900 0.30700700 0.97120200

C 7.62850600 -2.03735400 0.59988900

H 5.64192300 -2.83768200 0.59724200

C 8.47210600 -0.94736400 0.66948500

H 8.06517100 -3.01999200 0.43918400

N 6.02005100 1.64177200 1.39787600

N 8.49312800 1.44389100 1.22235100

S 7.35199500 2.55617400 1.54273900

O -1.29178700 1.17317700 1.14326400

O -1.74033200 -0.47419100 2.71843300

O 1.30910100 -2.00749200 -1.92895400

O 0.87424500 -3.65851900 -0.35364600

C -2.46150700 1.93660800 1.33740000

H -2.16201300 2.98419300 1.28765700

H -3.20248900 1.74563800 0.54974100

H -2.91565600 1.73984900 2.31719400

C -0.83790000 -0.01450500 3.70074700

H -1.31756700 -0.17844100 4.66645900

H 0.10405500 -0.57676800 3.67507700

H -0.62513100 1.05560800 3.58353300

C 0.40806500 -2.47137200 -2.91074200

H -0.53351500 -1.90837900 -2.88977400

H 0.88954700 -2.31198800 -3.87627700

H 0.19510800 -3.54091400 -2.78909200

C 2.04769000 -4.41452600 -0.55549000

H 2.79304900 -4.21756100 0.22652900

H 1.75552400 -5.46404200 -0.50290200

H 2.49398300 -4.21554400 -1.53827400

C -6.00324700 -1.69477800 -0.17871500

C -6.86354600 -2.81916000 -0.41292100

C -6.64070900 -0.49770800 0.08600000

C -8.29530400 -2.67571100 -0.36232600

C -8.03600200 -0.35135100 0.09921600

H -6.03596400 0.38761900 0.25522600

C -8.90154600 -1.40257700 -0.12776100

H -8.44772900 0.63095600 0.31642600

N -6.48053800 -4.07182000 -0.64883300

N -8.95381100 -3.80838100 -0.56994100

S -7.82876800 -4.96211400 -0.79810600

C 9.88968000 -1.15159200 0.51575800

H 10.23567400 -2.10600900 0.90551300

C -10.32589200 -1.30048600 -0.05595400

H -10.84126000 -2.20117200 0.28758200

C -12.16220200 1.87719100 -0.44753400

C -13.07428500 1.05796300 0.22143900

C -14.26959200 1.54189800 0.73102600

C -14.54767100 2.91104200 0.57894600

C -13.62024700 3.73986800 -0.07807200

C -12.43110600 3.23019800 -0.59895700

C -11.15874200 -0.24622200 -0.26751900

H -11.75087300 3.90842000 -1.09964900

C -12.53336500 -0.32565400 0.30201800

O -13.08202700 -1.28422400 0.77485400

C -11.01955200 1.05263300 -0.90082200

C -10.16407700 1.40523100 -1.90938500

C -9.26304900 0.48322100 -2.50756600

N -8.54020800 -0.25631100 -3.02114800

C -10.16914800 2.69723400 -2.50520700

N -10.15486500 3.74929400 -2.98128000

C 12.84533900 0.80665700 -0.39919300

C 11.84583200 1.58100200 -0.98868700

C 12.08402900 2.82268800 -1.53087600

C 13.37003900 3.34918000 -1.43140600

C 14.36766300 2.64471900 -0.73297700

C 14.10911800 1.37020900 -0.19078500

C 10.81095400 -0.32163300 -0.03026900

H 11.28356100 3.38107800 -2.00565000

C 10.53326300 0.89742000 -0.84243500

O 9.50090000 1.25395900 -1.34440800

C 12.26772800 -0.50127100 -0.04151300

C 12.96997400 -1.67045100 0.02463300

C 14.33551000 -1.78370600 -0.36099900

N 15.41483500 -1.92470200 -0.74519300

C 12.33966000 -2.91772400 0.30034000

N 11.82954700 -3.92786200 0.52880800

C -13.88982500 5.13503400 -0.21599900

N -14.08985000 6.26427200 -0.33181600

C -15.75664200 3.46154500 1.09354700

N -16.73359700 3.91016400 1.51001600

C 13.65160300 4.63038100 -1.99215600

N 13.85257700 5.66666900 -2.45544600

C 15.64634900 3.24537400 -0.54302900

N 16.67707200 3.73945400 -0.39337900

C -15.19968500 0.69412400 1.40415300

N -15.98630000 0.05972500 1.95801200

C 15.07802400 0.79209100 0.68014400

N 15.83276300 0.38855900 1.45274400

Table S5: Cartesian Co-ordinates of **IBRD4**

**Atoms X Y Z**

C 3.83746800 2.36315200 -1.45359300

C 4.51968200 1.74631000 -0.40187700

C 3.77412500 1.14198000 0.62865000

C 2.40605900 1.12269000 0.55575000

C 1.72814100 1.74221400 -0.50891400

C 2.45044800 2.37181400 -1.51100000

H 4.40578600 2.84942700 -2.23742800

H 4.28213500 0.74720200 1.50355600

H 1.94756400 2.88413400 -2.32397300

C 0.30554300 1.61936100 -0.24025200

C 1.40423200 0.60356500 1.58491500

C 0.09260300 1.02406000 0.94684300

C -1.00605900 2.03522500 -0.88129000

C -1.33000000 0.89986000 1.21452500

C -2.00800300 1.51586700 0.14765800

C -3.37544600 1.49197800 0.07186500

H -3.88190100 1.88009400 -0.80681700

C -2.05333700 0.27157100 2.21651200

H -1.55103600 -0.23961400 3.03062800

C -3.44058600 0.27770800 2.15751500

H -4.00828300 -0.21173200 2.93922100

C -4.12352400 0.88921400 1.10271800

C -5.58674200 0.93608700 1.04548900

C -6.42832500 -0.10915700 1.55304400

C -6.24195500 2.01888300 0.49818600

C -7.86314600 -0.04163800 1.41847900

C -7.64282500 2.10157000 0.39841900

H -5.65433900 2.86384300 0.15205400

C -8.48322200 1.07867200 0.78204500

H -8.08008100 3.00080300 -0.02827100

N -6.01932000 -1.20826200 2.18274600

N -8.49545700 -1.06115800 1.98390400

S -7.35203900 -2.04229600 2.58813100

O 1.32154800 -0.78910800 1.71454900

O 1.74824500 1.22830000 2.78544500

O -1.34618400 1.40671500 -2.08021200

O -0.92768900 3.42817200 -1.01334700

C 2.50457100 -1.45300000 2.09771300

H 2.22603500 -2.48469200 2.31664100

H 3.24640900 -1.45265000 1.28820200

H 2.94840100 -1.00318300 2.99524800

C 0.85191300 1.04815600 3.85915900

H 1.32455400 1.49005300 4.73731900

H -0.10191300 1.55935300 3.67678400

H 0.66090500 -0.01526500 4.05262700

C -0.44957100 1.58618900 -3.15428500

H 0.50483100 1.07664800 -2.97097300

H -0.92158600 1.14175500 -4.03141100

H -0.26057200 2.64955000 -3.34975100

C -2.11138200 4.08646300 -1.40373400

H -2.85689600 4.08580500 -0.59743000

H -1.83581400 5.11852100 -1.62482800

H -2.55018800 3.63236800 -2.30149600

C 5.98289800 1.69605600 -0.36182100

C 6.81121500 2.76114900 -0.84732100

C 6.64911900 0.61194000 0.16725000

C 8.24631400 2.66815300 -0.77486800

C 8.05310000 0.50837900 0.20766400

H 6.06642700 -0.22505300 0.54042500

C 8.88445900 1.49730900 -0.26487200

H 8.49082000 -0.38701500 0.64150400

N 6.39481600 3.91739400 -1.35786500

N 8.87658500 3.74101000 -1.23716200

S 7.72162000 4.78429000 -1.71235800

C -9.90871200 1.22173600 0.61338100

H -10.27161400 2.23614500 0.76498900

C 10.32120800 1.43758700 -0.20560100

H 10.82619900 2.38159300 0.01456200

C 12.15002600 -1.75210900 -0.20401000

C 13.08326800 -0.84454700 0.30717700

C 14.28909200 -1.24640500 0.83823900

C 14.56989100 -2.60369300 0.88033700

C 13.63671400 -3.52746400 0.38723700

C 12.43061200 -3.11126200 -0.16342200

C 11.15805000 0.38073200 -0.31018200

H 11.74638100 -3.86325400 -0.53688700

C 12.55674500 0.53194900 0.20008700

O 13.11475800 1.55246600 0.51062500

C 11.00135300 -1.00286100 -0.74560500

C 10.11086100 -1.48452600 -1.66593300

C 9.19686400 -0.65084600 -2.36809200

N 8.46547700 0.00702900 -2.97272900

C 10.09354400 -2.84974400 -2.06636200

N 10.05852200 -3.95943200 -2.38424000

C -12.78703600 -0.81411300 -0.44103500

C -11.71923900 -1.64199000 -0.79165300

C -11.89231400 -2.83921900 -1.44972100

C -13.18274500 -3.23669900 -1.76183800

C -14.26871400 -2.41915000 -1.41652500

C -14.08147500 -1.20928400 -0.76013200

C -10.80326800 0.26306100 0.26890500

H -11.03670900 -3.45225000 -1.71405200

C -10.43780400 -1.02842800 -0.38835300

O -9.34188500 -1.45330500 -0.65234900

C -12.26102500 0.38403700 0.23540300

C -13.02123700 1.38041400 0.78455300

C -12.47374900 2.49030400 1.48732400

N -12.05684600 3.40310900 2.05945600

C -14.44248200 1.39256700 0.71634600

N -15.59598000 1.42124300 0.66803600

Cl 16.07262600 -3.12923700 1.54823400

Cl -15.88255800 -2.89681300 -1.80546200

H -14.95467000 -0.61432000 -0.52484800

H 14.99837400 -0.51666100 1.21585200

Cl -13.42416100 -4.74009000 -2.57598000

Cl 13.96205800 -5.22250400 0.44890700

Table S6: Cartesian Co-ordinates of **IBRD5**

**Atoms X Y Z**

C -3.76825400 -2.38130900 -1.47335800

C -4.48358500 -1.74650500 -0.45458400

C -3.76957700 -1.09877900 0.57226900

C -2.40118500 -1.05541700 0.52599100

C -1.69000700 -1.69559200 -0.50452000

C -2.38026200 -2.36762700 -1.50144100

H -4.31130400 -2.90014800 -2.25405300

H -4.30181000 -0.68676800 1.42439600

H -1.85176700 -2.89490200 -2.28813300

C -0.27565200 -1.53757700 -0.21144400

C -1.42999300 -0.48196900 1.55571900

C -0.09870700 -0.89474000 0.95601000

C 1.05642700 -1.95663900 -0.80622200

C 1.31538400 -0.73423300 1.24866700

C 2.02730300 -1.38550000 0.22616700

C 3.39705500 -1.34900900 0.18661800

H 3.93610300 -1.77669400 -0.65365100

C 2.00257000 -0.04713200 2.23739800

H 1.47149200 0.49212900 3.01429300

C 3.39064700 -0.03140700 2.21046600

H 3.93308600 0.50439900 2.98030800

C 4.10671800 -0.68589500 1.20562400

C 5.57221000 -0.71958800 1.20102100

C 6.37825400 0.39027800 1.61608900

C 6.25851300 -1.84810400 0.80895400

C 7.81787300 0.32497700 1.56389800

C 7.66473700 -1.92536300 0.79179600

H 5.69345700 -2.73411700 0.53453300

C 8.47567400 -0.85794100 1.10564100

H 8.12896500 -2.86274400 0.49568300

N 5.92823700 1.55435600 2.07961500

N 8.41370100 1.41669200 2.02602200

S 7.23351500 2.45067500 2.44001500

O -1.38062000 0.91560300 1.64204000

O -1.78160600 -1.07594500 2.76990300

O 1.41638700 -1.36516400 -2.01860800

O 0.99998500 -3.35428000 -0.89222000

C -2.58330800 1.56203100 1.99190500

H -2.32998900 2.60333400 2.19572500

H -3.31113300 1.53151900 1.17021800

H -3.03263500 1.11969000 2.89028700

C -0.90864700 -0.84176900 3.85272000

H -1.38919500 -1.26231300 4.73703300

H 0.05801200 -1.33999900 3.70544500

H -0.74089600 0.23102200 4.01282900

C 0.54256800 -1.58752700 -3.10328500

H -0.41948900 -1.08083400 -2.95505400

H 1.02782000 -1.16790600 -3.98538300

H 0.36561500 -2.65826600 -3.26765500

C 2.20006700 -4.00536200 -1.24210600

H 2.92788400 -3.97777800 -0.42022400

H 1.94280400 -5.04555900 -1.44677200

H 2.65178000 -3.56405800 -2.13967300

C -5.94763100 -1.71915400 -0.44599500

C -6.75052000 -2.79965500 -0.94117200

C -6.64211300 -0.64091500 0.06009400

C -8.18834000 -2.72620600 -0.90280300

C -8.04722400 -0.55826300 0.07003700

H -6.08137900 0.20809300 0.43956700

C -8.85490200 -1.56164700 -0.41443500

H -8.50650300 0.33276900 0.49025600

N -6.30698000 -3.95406500 -1.43257200

N -8.79252000 -3.81122200 -1.37137200

S -7.61300300 -4.84215100 -1.81089900

C 9.91396900 -0.99447100 1.05668300

H 10.28849200 -1.96009400 1.39398600

C -10.29226100 -1.52274500 -0.38790600

H -10.78885700 -2.47683900 -0.19464300

C -12.16199500 1.63664100 -0.36829800

C -13.10201200 0.71334800 0.10954000

C -14.31396400 1.13173500 0.63330100

C -14.58262700 2.50476300 0.70649700

C -13.62470300 3.41569500 0.24293100

C -12.41951900 2.99558500 -0.30209500

C -11.14105800 -0.47482500 -0.49453300

H -11.71844000 3.74358500 -0.64938600

C -12.55082500 -0.66014300 -0.02399700

O -13.08290900 -1.70647200 0.23253100

C -10.99730700 0.91087000 -0.90774700

C -10.10503400 1.41670900 -1.81391400

C -9.16848300 0.60083500 -2.50727600

N -8.41771200 -0.04431100 -3.10179800

C -10.10165800 2.78283900 -2.21117800

N -10.07462200 3.89265300 -2.52946100

C 12.83137500 0.66531900 -0.35919300

C 11.81422500 1.50777500 -0.82026900

C 12.01647200 2.47879900 -1.77065200

C 13.28775800 2.60940300 -2.30597700

C 14.30602800 1.71200400 -1.95473000

C 14.07016200 0.71055800 -0.99832700

C 10.81562000 -0.08979700 0.61306000

H 11.19859500 3.11180000 -2.09830900

C 10.50829600 1.08370400 -0.26021000

O 9.43919500 1.53315500 -0.58034600

C 12.27549700 -0.18302500 0.70518600

C 12.94801800 -0.73059400 1.76171500

C 12.27335100 -1.48249600 2.76790000

N 11.75091600 -2.11013700 3.58427500

C 14.30983000 -0.46558700 2.07815000

N 15.38305700 -0.22677000 2.43010000

Cl -13.91907800 5.11420900 0.33519900

Cl -16.06840500 3.06564200 1.36202300

Cl -15.46903300 -0.01275000 1.19165000

Cl 13.57042900 3.85708500 -3.46560800

Cl 15.83763100 1.81382500 -2.72893400

Cl 15.24772800 -0.52491900 -0.76867300

Table S7: Cartesian Co-ordinates of **IBRD6**

**Atoms X Y Z**

C 3.89654300 2.14351200 -1.30098100

C 4.54848900 1.47674200 -0.26115800

C 3.77398500 0.83103300 0.72149700

C 2.40794400 0.82543100 0.61428900

C 1.76011900 1.49761700 -0.43710600

C 2.51130800 2.16515500 -1.39218300

H 4.48657500 2.65822300 -2.04957700

H 4.25806300 0.39370600 1.58982600

H 2.03258000 2.71850000 -2.19269300

C 0.33043700 1.37691000 -0.20538100

C 1.37855400 0.27294900 1.59773700

C 0.08587600 0.73426100 0.95028000

C -0.96303100 1.83134100 -0.85687100

C -1.34323300 0.61198600 1.18134900

C -1.99244200 1.27751400 0.12629000

C -3.35792500 1.26500800 0.01671700

H -3.84082200 1.69224000 -0.85719200

C -2.09383400 -0.05112800 2.13954900

H -1.61475700 -0.60012200 2.94284300

C -3.47910000 -0.03219400 2.04863600

H -4.06652100 -0.54628800 2.79883700

C -4.13390300 0.62405600 1.00319500

C -5.59556000 0.67657600 0.91337500

C -6.44952700 -0.38836100 1.35608200

C -6.23723700 1.78295100 0.39890300

C -7.88126000 -0.31021000 1.19536600

C -7.63586100 1.87331800 0.27208100

H -5.64137500 2.64095100 0.10246200

C -8.48588800 0.83780000 0.59425100

H -8.06213100 2.79107000 -0.12522900

N -6.05650400 -1.51587600 1.94464400

N -8.52774100 -1.35068600 1.70357300

S -7.39934900 -2.36194900 2.28631400

O 1.27921400 -1.12334000 1.66560300

O 1.70325900 0.84191900 2.83135700

O -1.28139400 1.25763600 -2.08906800

O -0.87059200 3.22820100 -0.92727700

C 2.44555200 -1.81498800 2.05073100

H 2.15140500 -2.85177800 2.21960600

H 3.20827100 -1.78956300 1.26129600

H 2.87011900 -1.40703700 2.97700100

C 0.78531500 0.62316300 3.87918800

H 1.24545100 1.02083100 4.78473700

H -0.16065600 1.15109400 3.70325700

H 0.58183800 -0.44585600 4.02158600

C -0.36229500 1.47806600 -3.13617100

H 0.58505500 0.95351500 -2.95818400

H -0.81995900 1.07747000 -4.04159200

H -0.16259300 2.54757200 -3.27969600

C -2.03861000 3.91226100 -1.32087600

H -2.80510800 3.88597300 -0.53499100

H -1.74912500 4.94989600 -1.49266300

H -2.45703000 3.49854200 -2.24731100

C 6.01052600 1.41999300 -0.18363200

C 6.85284800 2.51286200 -0.57392800

C 6.65986600 0.30285900 0.29350200

C 8.28458600 2.41483000 -0.45716500

C 8.06279500 0.19566700 0.37439200

H 6.06619900 -0.55578700 0.59317000

C 8.90632800 1.21399700 -0.00143100

H 8.48687400 -0.72528300 0.76592200

N 6.45321900 3.69834100 -1.02772700

N 8.92990100 3.51263600 -0.83183000

S 7.79043800 4.58311700 -1.28406700

C -9.90835600 0.99106100 0.40545000

H -10.27200700 1.99876100 0.59481200

C 10.34120200 1.14987800 0.11436400

H 10.83360000 2.06324100 0.45779900

C 12.15679300 -2.03362600 -0.15857300

C 13.06907100 -1.19836500 0.49441300

C 14.25281400 -1.66501000 1.03389800

C 14.51088500 -3.01313800 0.92207000

C 13.60280300 -3.86269900 0.28271400

C 12.42862300 -3.39482900 -0.27019100

C 11.17964500 0.10665600 -0.06652300

H 11.77109500 -4.10471900 -0.75785800

C 12.55412100 0.18533700 0.51993700

O 13.10157200 1.15883300 0.96960600

C 11.03628600 -1.21894500 -0.65983100

C 10.18194900 -1.58522500 -1.66297700

C 9.30166300 -0.66993400 -2.30401500

N 8.60009100 0.05474600 -2.86613800

C 10.17497800 -2.89734200 -2.21395300

N 10.15169500 -3.96527100 -2.65304400

C -12.77475200 -1.00802200 -0.75138900

C -11.70342000 -1.82511100 -1.11587400

C -11.86378700 -3.00873300 -1.80992000

C -13.14899900 -3.38362500 -2.13071600

C -14.23448200 -2.58030200 -1.77038800

C -14.07214000 -1.39446600 -1.08574200

C -10.79842000 0.04974700 0.00755400

H -11.01310800 -3.62184600 -2.08880100

C -10.42718200 -1.22060600 -0.68700700

O -9.32882100 -1.63561000 -0.95704400

C -12.25596700 0.17242900 -0.04119200

C -13.02388000 1.15740400 0.51804500

C -12.48832000 2.25450400 1.24928800

N -12.08136200 3.15715500 1.84424700

C -14.44410700 1.16905800 0.43047700

N -15.59690400 1.19853600 0.36775500

F 15.61842500 -3.53570100 1.41969300

F -15.44796500 -2.98504100 -2.10721000

F -13.38627900 -4.50691000 -2.78642100

F 13.89510000 -5.15039300 0.21576700

H -14.95879700 -0.82071200 -0.84665900

H 14.95295500 -0.99948100 1.52843000

**UV-Visible absorption spectra**

**Gaseous phase**

**Table S8:** Wave length, excitation energy and oscillator strength of **IBR**

| **NO** | **DFT λ (nm)** | **E(eV)** | ***f*** | **MO contributions** |
| --- | --- | --- | --- | --- |
| 1 | 569.362 | 2.178 | 1.684 | H→L (94%), H-1→L+1 (4%) |
| 2 | 511.634 | 2.423 | 0.020 | H→L+1 (96%) |
| 3 | 457.929 | 2.708 | 0.000 | H-3→L (49%), H-3→L+1 (32%), H-3→L+2 (14%) |
| 4 | 457.810 | 2.708 | 0.000 | H-2→L (43%), H-2→L+1 (38%), H-2→L+3 (14%) |
| 5 | 439.349 | 2.822 | 0.049 | H-1→L (87%), H-4→L+1 (9%) |
| 6 | 421.916 | 2.939 | 0.376 | H-4→L (21%), H-1→L+1 (70%), H→L (4%) |

MO=molecular orbital, HOMO=H, LUMO=L, ***f=*** oscillator strength

**Table S9:** Wave length, excitation energy and oscillator strength of **IBRD1**

| **NO** | **DFT λ (nm)** | **E(eV)** | ***f*** | **MO contributions** |
| --- | --- | --- | --- | --- |
| 1 | 617.913 | 2.007 | 1.410 | H→L (93%), H-1→L+1 (4%) |
| 2 | 562.210 | 2.205 | 0.088 | H→L+1 (96%) |
| 3 | 459.286 | 2.700 | 0.205 | H→L+2 (87%), H-1→L+3 (4%) |
| 4 | 447.516 | 2.771 | 0.028 | H-1→L (34%), H→L+3 (49%), H-2→L (2%), H-2→L+1 (4%), H-1→L+1 (2%), H→L+2 (2%) |
| 5 | 445.618 | 2.782 | 0.018 | H-1→L (45%), H→L+3 (40%), H-2→L+1 (7%) |
| 6 | 433.526 | 2.860 | 0.277 | H-2→L (15%), H-1→L+1 (68%), H-7→L+1 (2%), H→L (4%), H→L+3 (2%) |

MO=molecular orbital, HOMO=H, LUMO=L, ***f=*** oscillator strength

**Table S10:** Wave length, excitation energy and oscillator strength of **IBRD2**

| **NO** | **DFT λ (nm)** | **E(eV)** | ***f*** | **MO contributions** |
| --- | --- | --- | --- | --- |
| 1 | 690.989 | 1.794 | 1.267 | H→L (94%), H-1→L+1 (3%) |
| 2 | 626.879 | 1.978 | 0.186 | H→L+1 (97%) |
| 3 | 538.874 | 2.301 | 0.248 | H→L+2 (90%), H→L+3 (3%) |
| 4 | 522.303 | 2.374 | 0.023 | H→L+3 (92%), H→L+2 (3%) |
| 5 | 480.764 | 2.579 | 0.080 | H-1→L (79%), H-2→L (8%), H-2→L+1 (7%) |
| 6 | 463.752 | 2.674 | 0.329 | H-1→L+1 (73%), H-2→L (8%), H-2→L+1 (5%), H-1→L (4%), H→L (2%), H→L+4 (2%) |

MO=molecular orbital, HOMO=H, LUMO=L, ***f=*** oscillator strength

**Table S11:** Wave length, excitation energy and oscillator strength of **IBRD3**

| **NO** | **DFT λ (nm)** | **E(eV)** | ***f*** | **MO contributions** |
| --- | --- | --- | --- | --- |
| 1 | 734.069 | 1.689 | 1.145 | H→L (93%), |
| 2 | 661.320 | 1.875 | 0.201 | H→L+1 (95%), H→L (2%) |
| 3 | 577.181 | 2.148 | 0.223 | H→L+2 (92%), |
| 4 | 556.382 | 2.228 | 0.044 | H→L+3 (93%), H-1→L+3 (2%), H→L+2 (2%) |
| 5 | 501.372 | 2.473 | 0.128 | H-2→L (10%), H-1→L (80%), H-2→L+1 (2%), H→L (2%), H→L+4 (3%) |
| 6 | 478.113 | 2.593 | 0.379 | H-1→L+1 (63%), H→L+4 (16%), H-2→L+1 (8%), H-1→L (4%) |

MO=molecular orbital, HOMO=H, LUMO=L, ***f=*** oscillator strength

**Table S12:** Wave length, excitation energy and oscillator strength of **IBRD4**

| **NO** | **DFT λ (nm)** | **E(eV)** | ***f*** | **MO contributions** |
| --- | --- | --- | --- | --- |
| 1 | 641.441 | 1.933 | 1.364 | H→L (93%), H-1→L+1 (4%) |
| 2 | 583.126 | 2.126 | 0.139 | H→L+1 (97%), |
| 3 | 475.837 | 2.606 | 0.251 | H→L+2 (89%), H-1→L+3 (3%) |
| 4 | 462.490 | 2.681 | 0.012 | H→L+3 (84%), H-1→L (6%), H-1→L+2 (2%) |
| 5 | 456.866 | 2.714 | 0.071 | H-1→L (73%), H-2→L (4%), H-2→L+1 (9%), H→L+3 (7%) |
| 6 | 443.006 | 2.799 | 0.289 | H-2→L (12%), H-1→L+1 (71%), H-2→L+1 (2%), H-1→L (2%), H→L (3%) |

MO=molecular orbital, HOMO=H, LUMO=L, ***f=*** oscillator strength

**Table S13:** Wave length, excitation energy and oscillator strength of **IBRD5**

| **NO** | **DFT λ (nm)** | **E(eV)** | ***f*** | **MO contributions** |
| --- | --- | --- | --- | --- |
| 1 | 645.516 | 1.921 | 1.132 | H→L (91%), H-1→L (2%), H→L+1 (3%) |
| 2 | 574.373 | 2.159 | 0.319 | H→L+1 (92%), H→L (3%) |
| 3 | 484.162 | 2.561 | 0.226 | H→L+2 (82%), H-1→L+3 (4%), H→L+3 (9%) |
| 4 | 472.915 | 2.622 | 0.001 | H→L+3 (83%), H-1→L+2 (3%), H→L+2 (9%) |
| 5 | 461.028 | 2.689 | 0.103 | H-2→L (13%), H-1→L (78%), H→L (3%) |
| 6 | 434.225 | 2.855 | 0.306 | H-2→L+1 (11%), H-1→L+1 (74%), H-2→L (4%), H→L+1 (2%) |

MO=molecular orbital, HOMO=H, LUMO=L, ***f=*** oscillator strength

**Table S14:** Wave length, excitation energy and oscillator strength of **IBRD6**

| **NO** | **DFT λ (nm)** | **E(eV)** | ***f*** | **MO contributions** |
| --- | --- | --- | --- | --- |
| 1 | 630.001 | 1.968 | 1.295 | H→L (93%), H-1→L+1 (4%) |
| 2 | 575.680 | 2.154 | 0.129 | H→L+1 (97%), |
| 3 | 470.064 | 2.638 | 0.237 | H→L+2 (89%), H-1→L+3 (3%) |
| 4 | 456.647 | 2.715 | 0.010 | H→L+3 (85%), H-1→L (5%), H-1→L+2 (2%) |
| 5 | 450.720 | 2.751 | 0.058 | H-2→L+1 (10%), H-1→L (72%), H-2→L (4%), H→L+3 (7%) |
| 6 | 437.906 | 2.831 | 0.288 | H-2→L (13%), H-1→L+1 (71%), H-1→L (3%), H→L (3%) |

MO=molecular orbital, HOMO=H, LUMO=L, ***f=*** oscillator strength

**Solvent (Chloroform)**

**Table S15:** Wave length, excitation energy and oscillator strength of **IBR**

| **NO** | **DFT λ (nm)** | **E(eV)** | ***f*** | **MO contributions** |
| --- | --- | --- | --- | --- |
| 1 | 575.653 | 2.154 | 1.977 | H→L (92%), H-1→L+1 (6%) |
| 2 | 516.601 | 2.400 | 0.041 | H→L+1 (92%), H-1→L (6%) |
| 3 | 442.848 | 2.800 | 0.062 | H-2→L+1 (11%), H-1→L (82%), H→L+1 (6%) |
| 4 | 439.427 | 2.822 | 0.000 | H-4→L (47%), H-4→L+1 (33%), H-4→L+2 (10%), H-4→L+3 (7%) |
| 5 | 439.069 | 2.824 | 0.000 | H-3→L (42%), H-3→L+1 (38%), H-3→L+2 (8%), H-3→L+3 (8%) |
| 6 | 424.487 | 2.921 | 0.484 | H-2→L (25%), H-1→L+1 (69%), H→L (5%) |

MO=molecular orbital, HOMO=H, LUMO=L, ***f=*** oscillator strength

**Table S16:** Wave length, excitation energy and oscillator strength of **IBRD1**

| **NO** | **DFT λ (nm)** | **E(eV)** | ***f*** | **MO contributions** |
| --- | --- | --- | --- | --- |
| 1 | 619.580 | 2.001 | 1.627 | H→L (91%), H-1→L+1 (5%) |
| 2 | 566.112 | 2.190 | 0.082 | H→L+1 (93%), H-1→L (5%) |
| 3 | 455.942 | 2.719 | 0.206 | H→L+2 (81%), H-1→L (5%), H-1→L+3 (5%) |
| 4 | 450.311 | 2.753 | 0.027 | H-2→L+1 (12%), H-1→L (73%), H→L+1 (4%), H→L+2 (7%) |
| 5 | 443.482 | 2.796 | 0.007 | H→L+3 (84%), H-1→L+1 (2%), H-1→L+2 (6%) |
| 6 | 437.226 | 2.836 | 0.325 | H-2→L (18%), H-1→L+1 (68%), H→L (6%), H→L+3 (4%) |

MO=molecular orbital, HOMO=H, LUMO=L, ***f=*** oscillator strength

**Table S17:** Wave length, excitation energy and oscillator strength of **IBRD2**

| **NO** | **DFT λ (nm)** | **E(eV)** | ***f*** | **MO contributions** |
| --- | --- | --- | --- | --- |
| 1 | 694.045 | 1.786 | 1.457 | H→L (91%), H-1→L+1 (4%), H→L+2 (2%) |
| 2 | 635.490 | 1.951 | 0.158 | H→L+1 (94%), H-1→L (3%) |
| 3 | 541.936 | 2.288 | 0.304 | H→L+2 (89%), H-1→L+3 (4%), H→L (3%) |
| 4 | 527.413 | 2.351 | 0.016 | H→L+3 (92%), H-1→L+2 (3%), H→L+2 (2%) |
| 5 | 488.069 | 2.540 | 0.073 | H-2→L+1 (11%), H-1→L (78%), H-2→L (3%), H→L+1 (3%) |
| 6 | 473.114 | 2.621 | 0.297 | H-2→L (14%), H-1→L+1 (73%), H-1→L (3%), H→L (4%) |

MO=molecular orbital, HOMO=H, LUMO=L, ***f=*** oscillator strength

**Table S18:** Wave length, excitation energy and oscillator strength of **IBRD3**

| **NO** | **DFT λ (nm)** | **E(eV)** | ***f*** | **MO contributions** |
| --- | --- | --- | --- | --- |
| 1 | 745.635 | 1.663 | 1.299 | H→L (92%), H-1→L+1 (3%) |
| 2 | 679.179 | 1.826 | 0.181 | H→L+1 (95%), |
| 3 | 586.103 | 2.115 | 0.255 | H→L+2 (91%), H-1→L+2 (2%) |
| 4 | 567.148 | 2.186 | 0.049 | H→L+3 (92%), H-1→L+3 (3%) |
| 5 | 512.480 | 2.419 | 0.100 | H-1→L (80%), H-2→L (8%), H-2→L+1 (5%), H→L (3%) |
| 6 | 492.470 | 2.518 | 0.333 | H-1→L+1 (73%), H-2→L (6%), H-2→L+1 (7%), H→L (3%), H→L+1 (2%), H→L+4 (4%) |

MO=molecular orbital, HOMO=H, LUMO=L, ***f=*** oscillator strength

**Table S19:** Wave length, excitation energy and oscillator strength of **IBRD4**

| **NO** | **DFT λ (nm)** | **E(eV)** | ***f*** | **MO contributions** |
| --- | --- | --- | --- | --- |
| 1 | 643.005 | 1.928 | 1.572 | H→L (91%), H-1→L+1 (5%) |
| 2 | 588.551 | 2.107 | 0.127 | H→L+1 (93%), H-1→L (4%) |
| 3 | 473.511 | 2.618 | 0.268 | H→L+2 (87%), H-1→L+3 (5%) |
| 4 | 462.455 | 2.681 | 0.065 | H-2→L+1 (12%), H-1→L (75%), H→L+1 (4%), H→L+3 (2%) |
| 5 | 460.514 | 2.692 | 0.008 | H→L+3 (87%), H-1→L+2 (5%) |
| 6 | 448.942 | 2.762 | 0.313 | H-2→L (17%), H-1→L+1 (71%), H→L (5%) |

MO=molecular orbital, HOMO=H, LUMO=L, ***f=*** oscillator strength

**Table S20:** Wave length, excitation energy and oscillator strength of **IBRD5**

| **NO** | **DFT λ (nm)** | **E(eV)** | ***f*** | **MO contributions** |
| --- | --- | --- | --- | --- |
| 1 | 646.525 | 1.918 | 1.282 | H→L (89%), H-1→L (3%), H-1→L+1 (2%), H→L+1 (2%) |
| 2 | 579.663 | 2.139 | 0.347 | H→L+1 (90%), H-1→L+1 (3%), H→L (3%) |
| 3 | 482.429 | 2.570 | 0.252 | H→L+2 (81%), H-1→L+3 (4%), H→L+3 (8%) |
| 4 | 470.653 | 2.634 | 0.004 | H→L+3 (83%), H-1→L+2 (5%), H→L+2 (8%) |
| 5 | 465.633 | 2.663 | 0.110 | H-2→L (13%), H-1→L (76%), H-2→L+1 (3%), H→L (5%) |
| 6 | 441.319 | 2.809 | 0.295 | H-2→L+1 (12%), H-1→L+1 (73%), H-2→L (5%), H→L+1 (4%) |

MO=molecular orbital, HOMO=H, LUMO=L, ***f=*** oscillator strength

**Table S21:** Wave length, excitation energy and oscillator strength of **IBRD6**

| **NO** | **DFT λ (nm)** | **E(eV)** | ***f*** | **MO contributions** |
| --- | --- | --- | --- | --- |
| 1 | 628.182 | 1.974 | 1.533 | H→L (90%), H-1→L+1 (5%) |
| 2 | 577.288 | 2.148 | 0.100 | H→L+1 (93%), H-1→L (5%) |
| 3 | 465.354 | 2.664 | 0.245 | H→L+2 (87%), H-1→L+3 (5%) |
| 4 | 454.738 | 2.727 | 0.049 | H-2→L+1 (13%), H-1→L (71%), H→L+1 (4%), H→L+3 (5%) |
| 5 | 452.299 | 2.741 | 0.008 | H→L+3 (83%), H-1→L (5%), H-1→L+2 (5%) |
| 6 | 442.279 | 2.803 | 0.314 | H-2→L (19%), H-1→L+1 (70%), H→L (6%) |

MO=molecular orbital, HOMO=H, LUMO=L, ***f=*** oscillator strength. All out put files of entitled compounds were accomplished by Gaussian 09 version D.01 (<https://gaussian.com/g09citation/> )

**Frequency analysis**

Table S22: Calculated vibrational frequencies, intensities and their assignments of IBR

| ***Freq*** | ***I_IR_*** |  | **Vibrational assignments** |
| --- | --- | --- | --- |
| 3197 | 11 |  | υ(s) C-H_Ben_ |
| 3195 | 8 |  | υ(s) C-H_Ben_ |
| 3182 | 9 |  | υ(as) C-H_Ben_ + υ C-H_Ben_ |
| 3172 | 26 |  | υ(as) C-H_Ben_ + υ C-H_Ben_ |
| 3168 | 31 |  | υ(as) C-H_Ben_ + υ C-H_Ben_ |
| 3142 | 13 |  | υ C-H |
| 3127 | 17 |  | υ(s) + (as) C-H_CH3_ |
| 3125 | 28 |  | υ(s) + (as) C-H_CH3_ |
| 3122 | 9 |  | υ (as) C-H_CH3_ |
| 3074 | 26 |  | υ (as) C-H_CH3_ |
| 3063 | 27 |  | υ (as) C-H_CH3_ |
| 2999 | 55 |  | υ (s) C-H_CH3_ + υ C-O |
| 2992 | 46 |  | υ (s) C-H_CH3_ + υ C-O |
| 1833 | 48 |  | υ C-O_thiadin_ + υ C-C + υ (*w*) C-H_CH3_ |
| 1832 | 912 |  | υ C-O_thiadin_ + υ C-C + υ (*w*) C-H_CH3_ |
| 1671 | 50 |  | υ(ρ + δ) C-H_Ben_ + υ(ρ) C-H + υ C-C |
| 1666 | 224 |  | υ C-O_thiadin_ + υ C=C + υ(ρ) C-H |
| 1664 | 356 |  | υ C=C + υ(ρ) C-H |
| 1580 | 39 |  | υ(C=C-C=C_Ben_) + υ(δ) C-H_Ben_ |
| 1579 | 804 |  | υ(C=C-C=C_Ben_) + υ(δ) C-H_Ben_ + υC-C |
| 1547 | 57 |  | υ(as) C=N_thiadiazole_ + υ(ρ) C-H_Ben_ |
| 1521 | 168 |  | υ(as) C=N_thiadiazole_ + υ(ρ) C-H_Ben_ + υ(δ) C-H_CH3_ |
| 1496 | 40 |  | υ($\tau$ + δ) C-H_CH3_ |
| 1470 | 82 |  | υ($\tau$ + δ) C-H_CH3_ |
| 1445 | 169 |  | υC=S_thiadin_ + υC-N_thiadin_ + υ (*w*) C-H_CH3_ |
| 1444 | 215 |  | υC=S_thiadin_ + υC-N_thiadin_ + υ (*w*) C-H_CH3_ |
| 1436 | 8 |  | υ (*w*) C-H_CH3_ |
| 1376 | 73 |  | υC=S_thiadin_ + υC-N_thiadin_ + υ (*w*) C-H_CH3_ |
| 1337 | 256 |  | υC-N_thiadiazole_ + υ(δ) C-H_Ben_ + υ(ρ) C-H |
| 1327 | 170 |  | υC-N_thiadiazole_ + υ(δ) C-H_Ben_ + υC-S_thiadin_ |
| 1313 | 401 |  | υC=S_thiadin_ + υC-N_thiadin_ + υ (*w*) C-H_CH3_ |
| 1313 | 1182 |  | υC=S_thiadin_ + υC-N_thiadin_ + υ (*w +*$\tau$) C-H_CH3_ |
| 1297 | 233 |  | υ(C=C-C=C_Ben_) + υ(ρ) C-H_Ben_ + υC-O |
| 1284 | 56 |  | υ(ρ) C-H_Ben_ |
| 1232 | 186 |  | υ(ρ + δ) C-H_Ben_ + υ C-C |
| 1216 | 228 |  | υ (*w +*$\tau$) C-H_CH3_ + υC-O |
| 1152 | 542 |  | υC=S_thiadin_ + υ (*w +*$\tau$) C-H_CH3_  + υ(δ) C-H_Ben_ |
| 1152 | 220 |  | υC=S_thiadin_ + υ (*w +*$\tau$) C-H_CH3_  + υ(δ) C-H_Ben_ |
| 1149 | 454 |  | υC=S_thiadin_ + υ (*w +*$\tau$) C-H_CH3_  + υ(δ) C-H_Ben_ |
| 1137 | 230 |  | υ(δ) C-H_Ben_ |
| 1061 | 212 |  | υ(ρ) C-H_Ben_  + υ(ρ) C-H |
| 1019 | 66 |  | υ(ρ) C-H_Ben_  + υ C-O |
| 1004 | 39 |  | υC=S_thiadin_ + υC-S_thiadin_ + υC-N_thiadin_ |
| 964 | 28 |  | υ(τ) C-H_Ben_ + υC-N |
| 963 | 96 |  | υ(τ) C-H_Ben_ + υC-N + υC-S_thiadin_ |
| 916 | 12 |  | υ(γ) C-H_Ben_ |
| 896 | 61 |  | υ C-S_thiadiazole_ |
| 839 | 40 |  | υ(*w*) C-H_Ben_ |
| 835 | 47 |  | υ(*w*) C-H_Ben_ |
| 776 | 37 |  | υ(τ) C-H_Ben_ + υ(γ) C-H_Ben_ |

Table S23: Calculated vibrational frequencies, intensities and their assignments of IBRD1

| ***Freq*** | ***I_IR_*** |  | **Vibrational assignments** |
| --- | --- | --- | --- |
| 3234 | 5 |  | υ(s) C-H_Ben_ |
| 3221 | 5 |  | υ(s) C-H_Ben_ |
| 3218 | 1.9 |  | υ C-H_Ben_ |
| 3213 | 2 |  | υ C-H_Ben_ |
| 3189 | 31 |  | υ(as) C-H_Ben_ |
| 3185 | 9 |  | υ C-H |
| 3180 | 5.5 |  | υ(s) + υ(as) C-H |
| 3127 | 18 |  | υ(s) + υ(as) C-H_CH3_ |
| 3126 | 20 |  | υ(s) + υ(as) C-H_CH3_ |
| 3116 | 2 |  | υ C-H |
| 3076 | 48 |  | υ(as) C-H_CH3_ |
| 3063 | 28 |  | υ(as) C-H_CH3_ |
| 3003 | 32 |  | υ(s) C-H_CH3_ |
| 2993 | 41 |  | υ(s) C-H_CH3_ |
| 2344 | 100 |  | υ(s) C-N |
| 2342 | 100 |  | υ(s) C-N |
| 2333 | 28 |  | υ(s) C-N |
| 1841 | 316 |  | υ C=O |
| 1834 | 237 |  | υ C=O |
| 1670 | 7 |  | υ C=C |
| 1669 | 140 |  | υ (C=C-C=C_Ben_) + υ( δ) C-H_Ben_ |
| 1666 | 390 |  | υ (C=C-C=C_Ben_) + υ( ρ) C-H_Ben_ |
| 1661 | 809 |  | υ (C=C-C=C_Ben_) + υ( ρ) C-H_Ben_ |
| 1655 | 2.7 |  | υ (C=C-C=C_pe_) + υ( ρ) C-H |
| 1654 | 27 |  | υ C=C + υ( ρ) C-H |
| 1637 | 8 |  | υ (C=C-C=C_pe_) + υ( ρ) C-H |
| 1633 | 36 |  | υ (C=C-C=C_Ben_) + υ( ρ) C-H_Ben_ |
| 1625 | 121 |  | υ( ρ) C-H_Ben_ |
| 1612 | 78 |  | υ (C=C-C=C_pe_) + υ C=C |
| 1606 | 200 |  | υ (C=C-C=C_pe_) + υ C=C + υ( ρ) C-H_Ben_ |
| 1599 | 10 |  | υ (C=C-C=C_Ben_) + υ( ρ) C-H_Ben_ |
| 1570 | 183 |  | υ (C=C-C=C_Ben_) + υ( δ) C-H_Ben_ |
| 1563 | 4727 |  | υ C=N + υ (C=C-C=C_Ben_) + υ( δ) C-H_Ben_ |
| 1544 | 837 |  | υ C=N + υ( ρ) C-H_Ben_ |
| 1517 | 413 |  | υ (C=C-C=C_Ben_) + υ( ρ) C-H_Ben_ |
| 1497 | 82 |  | υ( δ) C-H_CH3_ + υ( ρ) C-H_Ben_ |
| 1496 | 52 |  | υ( δ) C-H_CH3_ |
| 1452 | 12 |  | β(C=C-C=C_Ben_) |
| 1443 | 10 |  | υ(w) C-H_CH3_ |
| 1430 | 373 |  | υ( ρ) C-H + υ C=N + υ C=C |
| 1427 | 580 |  | υ( ρ) C-H + υ (C=C-C=C_Ben_) + υ C=N |
| 1415 | 208 |  | υ (C=C-C=C_Ben_) + υ( ρ) C-H_Ben_ |
| 1381 | 203 |  | υ( ρ) C-H_Ben_υ( ρ) C-H |
| 1359 | 7 |  | υ C=N |
| 1354 | 29 |  | υ( ρ) C-H |
| 1330 | 90 |  | υ( ρ) C-H_Ben_ + υ C=N |
| 1301 | 74 |  | υ (C=C-C=C_Ben_) + υ( ρ) C-H_Ben_ |
| 1290 | 79 |  | υ( δ) C-H_Ben_ |
| 1277 | 216 |  | υ (C=C-C=C_Ben_) + υ( ρ) + υ( δ) C-H_Ben_ |
| 1236 | 114 |  | υ(τ) + υ(w) C-H_CH3_ |
| 1216 | 249 |  | υ(τ) + υ(w) C-H_CH3_ |
| 1185 | 19 |  | υ( ρ) C-H_Ben_ |
| 1176 | 34 |  | υ(τ)C-H_CH3_ + υ( δ) C-H_Ben_ |
| 1171 | 80 |  | υ( ρ) C-H_Ben_ |
| 1134 | 264 |  | υ C-O |
| 970 | 10 |  | υ(τ)C-H_Ben_ |
| 965 | 9 |  | υ(τ)C-H_Ben_ |
| 917 | 113 |  | υ(γ)C-H |
| 907 | 13 |  | υ(γ)C-H |
| 904 | 28 |  | υ N-S |
| 897 | 36 |  | υ(γ)C-H_Ben_ |
| 892 | 18 |  | υ N-S |
| 880 | 10 |  | υ N-S |
| 872 | 20 |  | υ N-S + υ (C=C-C=C_pe_) |
| 860 | 23 |  | υ(w) C-H_Ben_ |
| 855 | 8 |  | υ(w) C-H_Ben_ |
| 774 | 18 |  | β(C=C-C=C_pe_) |
| 766 | 35 |  | υ(γ)(C=C-C=C_Ben_) |

Table S24: Calculated vibrational frequencies, intensities and their assignments of IBRD2

| ***Freq*** | ***I_IR_*** |  | **Vibrational assignments** |
| --- | --- | --- | --- |
| 3222 | 1 |  | υ(s) C-H_Ben_ |
| 3220 | 2 |  | υ(s) C-H_Ben_ |
| 3207 | 6 |  | υ C-H_Ben_ |
| 3205 | 7 |  | υ C-H_Ben_ |
| 3187 | 12 |  | υ(as) C-H_Ben_ |
| 3159 | 7 |  | υ C-H |
| 3157 | 16 |  | υ(s) + υ(as) C-H |
| 3133 | 18 |  | υ(s) + υ(as) C-H_CH3_ |
| 3131 | 23 |  | υ(s) + υ(as) C-H_CH3_ |
| 3113 | 3 |  | υ C-H |
| 3076 | 26 |  | υ(as) C-H_CH3_ |
| 3070 | 33 |  | υ(as) C-H_CH3_ |
| 3001 | 38 |  | υ(s) C-H_CH3_ |
| 2998 | 61 |  | υ(s) C-H_CH3_ |
| 2365 | 2 |  | υ(s) C-N |
| 2360 | 8 |  | υ(s) C-N |
| 2347 | 82 |  | υ(s) C-N |
| 1842 | 341 |  | υ C=O |
| 1832 | 191 |  | υ C=O |
| 1668 | 6 |  | υ C=C |
| 1667 | 89 |  | υ (C=C-C=C_Ben_) + υ( δ) C-H_Ben_ |
| 1661 | 368 |  | υ (C=C-C=C_Ben_) + υ( ρ) C-H_Ben_ |
| 1660 | 132 |  | υ (C=C-C=C_Ben_) + υ( ρ) C-H_Ben_ |
| 1656 | 65 |  | υ (C=C-C=C_pe_) + υ( ρ) C-H |
| 1652 | 1060 |  | υ C=C + υ( ρ) C-H |
| 1642 | 628 |  | υ (C=C-C=C_pe_) + υ( ρ) C-H |
| 1633 | 59 |  | υ (C=C-C=C_Ben_) + υ( ρ) C-H_Ben_ |
| 1622 | 63 |  | υ( ρ) C-H_Ben_ |
| 1612 | 186 |  | υ (C=C-C=C_pe_) + υ C=C |
| 1607 | 84 |  | υ (C=C-C=C_pe_) + υ C=C + υ( ρ) C-H_Ben_ |
| 1603 | 77 |  | υ (C=C-C=C_Ben_) + υ( ρ) C-H_Ben_ |
| 1570 | 183 |  | υ (C=C-C=C_Ben_) + υ( δ) C-H_Ben_ |
| 1563 | 4727 |  | υ C=N + υ (C=C-C=C_Ben_) + υ( δ) C-H_Ben_ |
| 1544 | 837 |  | υ C=N + υ( ρ) C-H_Ben_ |
| 1517 | 413 |  | υ (C=C-C=C_Ben_) + υ( ρ) C-H_Ben_ |
| 1497 | 82 |  | υ( δ) C-H_CH3_ + υ( ρ) C-H_Ben_ |
| 1496 | 52 |  | υ( δ) C-H_CH3_ |
| 1452 | 12 |  | β(C=C-C=C_Ben_) |
| 1443 | 10 |  | υ(w) C-H_CH3_ |
| 1430 | 373 |  | υ( ρ) C-H + υ C=N + υ C=C |
| 1425 | 580 |  | υ( ρ) C-H + υ (C=C-C=C_Ben_) + υ C=N |
| 1416 | 208 |  | υ (C=C-C=C_Ben_) + υ( ρ) C-H_Ben_ |
| 1384 | 304 |  | υ( ρ) C-H_Ben_υ( ρ) C-H |
| 1362 | 181 |  | υ C=N |
| 1353 | 177 |  | υ( ρ) C-H |
| 1329 | 280 |  | υ( ρ) C-H_Ben_ + υ C=N |
| 1302 | 100 |  | υ (C=C-C=C_Ben_) + υ( ρ) C-H_Ben_ |
| 1289 | 112 |  | υ( δ) C-H_Ben_ |
| 1287 | 226 |  | υ (C=C-C=C_Ben_) + υ( ρ) + υ( δ) C-H_Ben_ |
| 1234 | 268 |  | υ(τ) + υ(w) C-H_CH3_ |
| 1215 | 252 |  | υ(τ) + υ(w) C-H_CH3_ |
| 1189 | 258 |  | υ( ρ) C-H_Ben_ |
| 1177 | 74 |  | υ(τ)C-H_CH3_ + υ( δ) C-H_Ben_ |
| 1175 | 248 |  | υ( ρ) C-H_Ben_ |
| 1135 | 314 |  | υ C-O |
| 970 | 146 |  | υ(τ)C-H_Ben_ |
| 968 | 122 |  | υ(τ)C-H_Ben_ |
| 926 | 195 |  | υ(γ)C-H |
| 910 | 73 |  | υ(γ)C-H |
| 904 | 25 |  | υ N-S |
| 893 | 99 |  | υ(γ)C-H_Ben_ |
| 891 | 5 |  | υ N-S |
| 882 | 10 |  | υ N-S |
| 878 | 25 |  | υ N-S + υ (C=C-C=C_pe_) |
| 859 | 44 |  | υ(w) C-H_Ben_ |
| 841 | 122 |  | υ(w) C-H_Ben_ |
| 769 | 77 |  | β(C=C-C=C_pe_) |
| 753 | 2 |  | υ(γ)(C=C-C=C_Ben_) |

Table S25: Calculated vibrational frequencies, intensities and their assignments of IBRD3

| ***Freq*** | ***I_IR_*** | **Vibrational assignments** |
| --- | --- | --- |
| 3228 | 8.5 | $\upsilon$ (C-H) |
| 3213 | 2 | υ(s)C-H(_Ben_) |
| 3191 | 8 | υ(as)C-H(_Ben_) |
| 3178 | 4 | *w*C-H(_BTD_) + υC-H(_Ben_) |
| 3165 | 14 | υ(as) C-H(_BTD_) |
| 3127 | 15 | υ(as)C-H(_CH3_) |
| 3126 | 31 | $\rho$C-H(_CH3_) |
| 3076 | 29 | υ(as)C-H(_CH3_) |
| 3003 | 89 | $\upsilon$ (C-H) |
| 3002 | 15 | υ(as) C-H(_CH3_) |
| 2990 | 43 | $\upsilon$ + *w* C-H(_CH2_) |
| 2368 | 1 | $\upsilon$ (C=N) |
| 2347 | 35 | *w* (C=N) |
| 1842 | 313 | $\upsilon$ (C=O) |
| 1840 | 175 | *w* (C=O) |
| 1668 | 125 | $\delta$ C-H(_Ben_) |
| 1667 | 11 | $\upsilon$ (C=C-C=C_TBHI_) + (δ)C-H(_TBHI_) |
| 1656 | 884 | $\upsilon$ (C=C-C=C_Ben_) |
| 1655 | 14 | $\upsilon$ (C=C-C=C_Ben_) + (δ + ρ)C-H(_Ben_) |
| 1620 | 28 | $\rho$C-H(_BTD_) + $\upsilon$ (C=C _BTD_) + $\upsilon$ (C=C) |
| 1608 | 3.7 | (ρ) C-H(_BTD_) |
| 1594 | 13 | $(\upsilon$ +ρ)C-H(_TBHI_) + (ρ) C-H(_BTD_) |
| 1571 | 1055 | $(\upsilon$ +ρ )C-H(_BTD_) |
| 1545 | 396 | (δ + ρ)C-H(_BTD_) +$\upsilon$ (C=C=N-C_BTD_) |
| 1496 | 4.6 | (δ + ρ) C-H(_CH3_) |
| 1357 | 129 | $\upsilon$ (C=C-C=C_CMOIC_) + (δ + ρ)C-H(_CMOIC_) |
| 1352 | 231 | $(\upsilon$ +ρ)C-H(_CMOIC_) + (ρ) C-H(_BTD_) |
| 1292 | 189 | (δ + ρ)C-H(_TBHI_) |
| 1194 | 74 | (δ + ρ)C-H(_CMOIC_) |
| 1102 | 17 | (δ + ρ)C-H(_BTD_) +$\upsilon$ (C=C=N-C_CMOIC_) |
| 1040 | 62 | δ C-H(_BTD_) + $(\upsilon$ +ρ)C-H(_CMOIC_) |
| 958 | 578 | (*w*) C-H |
| 867 | 12 | (*w*) C-H _(TBHI)_ |
| 774 | 44 | ($\tau$) C-H(_TBHI_) |
| 635 | 7 | ($\tau$) C-H_(CMOIC)_ |
| 509 | 4 | (ρ) C-H |
| 440 | 50 | ($\tau+\rho$) C-H(_CMOIC_) |
| 335 | 10 | (ρ) C-H + (ρ) C=O |
| 200 | 6 | ($\tau$) C-H(_CH3_) |
| 131 | 6 | (ρ) C-N |
| 114 | 5.8 | (δ +ρ) C-N(_CMOIC_) + (ρ) C-H |
| 80 | 2 | ($\tau$) C-N |
| 44 | 2.1 | (δ + ρ +γ)C-H(_CMOIC_) + (ρ) C-H(_BTD_) |
| 11 | 0.136 | (*w* + ρ)C-H(_CMOIC_) + (ρ) C-H(_BTD_) + (*w*)C-H(_TBHI_) |

Table S26: Calculated vibrational frequencies, intensities and their assignments of IBRD4

| ***Freq*** | ***I_IR_*** | **Vibrational assignments** |
| --- | --- | --- |
| 3220 | 1.5 | υ C-H_ben_ |
| 3213 | 7 | υ(s) C-H_Ben_ |
| 3212 | 2 | υ(as) C-H_Ben_ |
| 3181 | 18 | υ +υ(as) C-H_Ben_ |
| 3179 | 2 | υ +υ(s) C-H_Ben_ |
| 3167 | 14 | υ(as) C-H_Ben_ |
| 3156 | 22 | υ(as) C-H_Ben_ |
| 3130 | 21 | υ(s) + (as) C-H_CH3_ |
| 3129 | 27 | υ(s) + (as) C-H_CH3_ |
| 3125 | 17 | υ(s) + (as) C-H_CH3_ |
| 3115 | 3 | υC-H |
| 3075 | 25 | υ(as) C-H_CH3_ |
| 3074 | 24 | υ(as) C-H_CH3_ |
| 3069 | 36 | υ(s)+ (as) C-H_CH3_ |
| 3000 | 41 | υ (s) C-H_CH3_ |
| 2998 | 44 | υ (s) C-H_CH3_ |
| 2997 | 42 | υ (s) C-H_CH3_ |
| 2345 | 91 | υ C ≡ N |
| 2335 | 24 | υ C ≡ N |
| 2333 | 25 | υ C ≡ N |
| 1841 | 323 | υ C=O |
| 1832 | 240 | υ C=O |
| 1670 | 311 | υ (C=C-C=C_Ben_) + υ(δ + ρ)C-H_Ben_ |
| 1654 | 899 | υ(δ + ρ)C-H_Ben_ |
| 1642 | 914 | υ(δ + ρ)C-H_Ben_ |
| 1636 | 18 | υ C=N + υ(ρ) C-H_ben_ |
| 1635 | 29 | υ (C=C-C=C_Ben_) + υ( ρ)C-H_Ben_ |
| 1625 | 88 | υ C=N + υ(ρ) C-H_ben_ |
| 1614 | 19 | υ C=C + υ(δ +ρ) C-H_ben_ |
| 1602 | 176 | υ (C=C-C=C_Ben_) + υ(ρ)C-H_Ben_ |
| 1598 | 14 | υ C=C + υ(δ ) C-H_ben_ |
| 1570 | 2987 | υ(δ + w) C-H_Ben_ |
| 1547 | 76 | υ(ρ+ w) C-H_Ben_ |
| 1546 | 210 | υ(ρ + w) C-H_Ben_+υ(δ ) C-H_CH3_ |
| 1456 | 3.3 | υ(δ + w)C-H_Ben_ |
| 1431 | 227 | υ(w + τ) C-H_CH3_ |
| 1426 | 18 | υ(ρ)C-H_Ben_ |
| 1424 | 159 | υ(δ+ ρ)C-H_Ben_ + υ C=N |
| 1404 | 7 | υ(ρ) C-H |
| 1376 | 30 | υ(δ+ ρ)C-H_Ben_ + υ C=N |
| 1359 | 53 | υ(ρ) C-H_Ben_ |
| 1337 | 287 | υ (C=C-C=C_Ben_)+υ(ρ) C-H_Ben_ |
| 1329 | 197 | υ(δ+ ρ)C-H_Ben_ + υ C=N |
| 1244 | 154 | υ(δ+ ρ)C-H_Ben_ |
| 1236 | 210 | υ(δ+ ρ)C-H_Ben_ |
| 1231 | 397 | υ( ρ)C-H_Ben_+ υ(w ) C-H_CH3_ |
| 1225 | 72 | υ( ρ)C-H_Ben_+ υ (C=C-C=C_Ben_ |
| 1177 | 54 | υ( ρ)C-H_Ben_ |
| 1177 | 83 | υ(δ+ ρ)C-H_Ben_+ υ(w+ τ ) C-H_CH3_ |
| 1134 | 48 | υ(δ )C-H_Ben_ + υ C=O |
| 1023 | 112 | υ(δ )C-H_Ben_+ υ(w ) C-H_CH3_ |
| 1021 | 3 | υ( ρ)C-H_Ben_ + υ(ρ) C-H |
| 1015 | 88 | υ(w+ τ ) C-H_CH3_ |
| 982 | 37 | υC-Cl |
| 972 | 43 | υ( τ ) C-H_ben_ |

Table S27: Calculated vibrational frequencies, intensities and their assignments of IBRD5

| ***Freq*** | ***I_IR_*** | **Vibrational assignments** |
| --- | --- | --- |
| 3214 | 9 | υ C-H_ben_ |
| 3213 | 3 | υ(s) C-H_Ben_ |
| 3182 | 15 | υ(as) C-H_Ben_ |
| 3181 | 14 | υ +υ(as) C-H_Ben_ |
| 3171 | 8 | υ +υ(s) C-H_Ben_ |
| 3160 | 17 | υ(as) C-H_Ben_ |
| 3156 | 27 | υ(as) C-H_Ben_ |
| 3131 | 21 | υ(s) + (as) C-H_CH3_ |
| 3130 | 25 | υ(s) + (as) C-H_CH3_ |
| 3125 | 22 | υ(s) + (as) C-H_CH3_ |
| 3115 | 3 | υC-H |
| 3073 | 26 | υ(as) C-H_CH3_ |
| 3073 | 28 | υ(as) C-H_CH3_ |
| 3069 | 18 | υ(s)+ (as) C-H_CH3_ |
| 2999 | 76 | υ (s) C-H_CH3_ |
| 2998 | 74 | υ (s) C-H_CH3_ |
| 2997 | 42 | υ (s) C-H_CH3_ |
| 2348 | 49 | υ C ≡ N |
| 2346 | 28 | υ C ≡ N |
| 2335 | 25 | υ C ≡ N |
| 1843 | 286 | υ C=O |
| 1837 | 232 | υ C=O |
| 1670 | 117 | υ (C=C-C=C_Ben_) + υ(δ + ρ)C-H_Ben_ |
| 1669 | 118 | υ(δ + ρ)C-H_Ben_ |
| 1664 | 818 | υ(δ + ρ)C-H_Ben_ |
| 1660 | 1244 | υ C=N + υ(ρ) C-H_ben_ |
| 1636 | 29 | υ (C=C-C=C_Ben_) + υ( ρ)C-H_Ben_ |
| 1625 | 110 | υ C=N + υ(ρ) C-H_ben_ |
| 1606 | 190 | υ C=C + υ(δ +ρ) C-H_ben_ |
| 1584 | 125 | υ (C=C-C=C_Ben_) + υ(ρ)C-H_Ben_ |
| 1579 | 583 | υ C=C + υ(δ ) C-H_ben_ |
| 1572 | 2205 | υ(δ + w) C-H_Ben_ |
| 1546 | 110 | υ(ρ+ w) C-H_Ben_ |
| 1517 | 204 | υ(ρ + w) C-H_Ben_+υ(δ ) C-H_CH3_ |
| 1497 | 34 | υ(δ + w)C-H_Ben_ |
| 1485 | 19 | υ(w + τ) C-H_CH3_ |
| 1435 | 54 | υ(ρ)C-H_Ben_ |
| 1430 | 546 | υ(δ+ ρ)C-H_Ben_ + υ C=N |
| 1406 | 46 | υ(ρ) C-H |
| 1378 | 35 | υ(δ+ ρ)C-H_Ben_ + υ C=N |
| 1378 | 59 | υ(ρ) C-H_Ben_ |
| 1337 | 287 | υ (C=C-C=C_Ben_)+υ(ρ) C-H_Ben_ |
| 1329 | 406 | υ(δ+ ρ)C-H_Ben_ + υ C=N |
| 1290 | 72 | υ(δ+ ρ)C-H_Ben_ |
| 1270 | 181 | υ(δ+ ρ)C-H_Ben_ |
| 1245 | 153 | υ( ρ)C-H_Ben_+ υ(w ) C-H_CH3_ |
| 1235 | 412 | υ( ρ)C-H_Ben_+ υ (C=C-C=C_Ben_ |
| 1179 | 182 | υ( ρ)C-H_Ben_ |
| 1177 | 63 | υ(δ+ ρ)C-H_Ben_+ υ(w+ τ ) C-H_CH3_ |
| 1134 | 48 | υ(δ )C-H_Ben_ + υ C=O |
| 1096 | 18 | υ(δ )C-H_Ben_+ υ(w ) C-H_CH3_ |
| 1042 | 163 | υ( ρ)C-H_Ben_ + υ(ρ) C-H |
| 1033 | 119 | υ(w+ τ ) C-H_CH3_ |
| 990 | 23 | υC-Cl |
| 973 | 7 | υ( τ ) C-H_ben_ |

Table S28: Calculated vibrational frequencies, intensities and their assignments of IBRD6

| ***Freq*** | ***I_IR_*** |  | **Vibrational assignments** |
| --- | --- | --- | --- |
| 3220 | 2 |  | υ C-H_Ben_ |
| 3217 | 3 |  | υ(s) C-H_Ben_ |
| 3213 | 10 |  | υ C-H_Ben_ |
| 3187 | 15 |  | υ(as) C-H_Ben_ |
| 3169 | 10 |  | υ(s) C-H_Ben_ |
| 3156 | 21 |  | υ(as) C-H_Ben_ |
| 3156 | 24 |  | υ(as) C-H_Ben_ |
| 3128 | 24 |  | υ(s) + υ(as) C-H_Ben_ |
| 3067 | 22 |  | υ(as) C-H_CH3_ |
| 3001 | 48 |  | υ(s) C-H_CH3_ |
| 2997 | 61 |  | υ(s) C-H_CH3_ |
| 2345 | 93 |  | υ(s) C-N |
| 2334 | 27 |  | υ(s) C-N |
| 1841 | 331 |  | υ C=O |
| 1833 | 226 |  | υ C=O |
| 1676 | 742 |  | υ C=C + υ( ρ) C-H |
| 1668 | 188 |  | υ (C=C-C=C_Ben_) + υ( δ) + υ( ρ) C-H_Ben_ |
| 1667 | 195 |  | υ (C=C-C=C_Ben_) + υ( ρ) C-H_Ben_ |
| 1666 | 207 |  | υ (C=C-C=C_Ben_) |
| 1657 | 244 |  | υ C=C |
| 1657 | 618 |  | υ C=C + υ( ρ) C-H |
| 1639 | 37 |  | υ (C=C-C=C_Ben_) |
| 1625 | 63 |  | υ( ρ) C-H_Ben_ |
| 1610 | 153 |  | υ( ρ) C-H_Ben_ + υ C=C |
| 1609 | 299 |  | υ C=C |
| 1580 | 341 |  | υ (C=C-C=C_Ben_) + υ( δ) C-H_Ben_ |
| 1573 | 2443 |  | υ (C=C-C=C_Ben_) + υ( δ) C-H_Ben_ |
| 1546 | 152 |  | υ (C=C-C=C_Ben_) + υ C-N |
| 1541 | 379 |  | υ C-F + υ( ρ) C-H_Ben_ |
| 1535 | 422 |  | υ C-F + υ( ρ) C-H_Ben_ |
| 1518 | 232 |  | υ (C=C-C=C_Ben_) |
| 1500 | 36 |  | υ( δ) C-H_CH3_ |
| 1493 | 21 |  | υ (C=C-C=C_Ben_) |
| 1488 | 25 |  | υ( δ) C-H_CH3_ |
| 1443 | 9 |  | υ(w) C-H_CH3_ |
| 1430 | 209 |  | υ( ρ) C-H |
| 1418 | 109 |  | υ( ρ) C-H_Ben_ |
| 1406 | 207 |  | υ( ρ) C-H |
| 1389 | 452 |  | υ (C=C-C=C_Ben_) |
| 1389 | 1215 |  | υ (C=C-C=C_Ben_) |
| 1349 | 203 |  | υ( ρ) C-H_Ben_ + υ( ρ) C-H |
| 1341 | 169 |  | υ (C=C-C=C_Ben_) |
| 1291 | 105 |  | υ( ρ) C-H_Ben_ |
| 1271 | 83 |  | υ( ρ) C-H |
| 1233 | 252 |  | υ(τ) + υ(w) C-H_CH3_ |
| 1215 | 244 |  | υ(τ) + υ(w) C-H_CH3_ |
| 1154 | 156 |  | υ( δ) C-H_Ben_ |
| 1141 | 115 |  | υ( δ) C-H_Ben_ + υ C=O |
| 1135 | 317 |  | υ C-O |
| 1103 | 82 |  | υ( δ) C-H_Ben_ |
| 1013 | 36 |  | υ( ρ) C-H_Ben_ |
| 970 | 21 |  | υ(τ)C-H_Ben_ |
| 966 | 43 |  | υ(τ)C-H_Ben_ |
| 948 | 196 |  | υ(γ)C-H |
| 926 | 134 |  | υ(γ)C-H |
| 909 | 30 |  | υ(γ)C-H_Ben_ |
| 903 | 50 |  | υ N-S |
| 881 | 40 |  | υ N-S |
| 847 | 112 |  | υ(w) C-H_Ben_ |
| 801 | 16 |  | υ(τ) + υ(w) C-H_CH3_ |
| 764 | 80 |  | υ C-F |
| 704 | 6 |  | β(C=C-C=C_Ben_) |

*Freq*=Frequencies are given in cm^-1^, $\upsilon$ =stretching, $\beta$=in-plane bending, γ=out-plane bending $\delta$=scissoring, $\rho$=rocking, *w*= wagging, s =symmetric, as=asymmetric,$\tau$=twisting, Ben=benzene ring, 2-thioxo-thiazolidin-4-one = thiadin, 1,2,5-thiadiazole = thiadiazole.


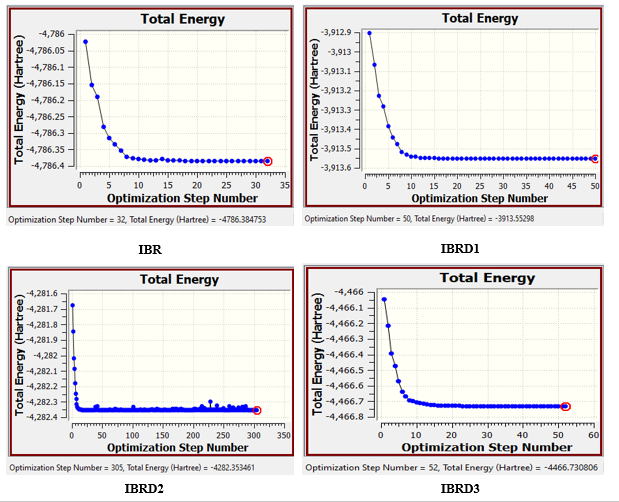


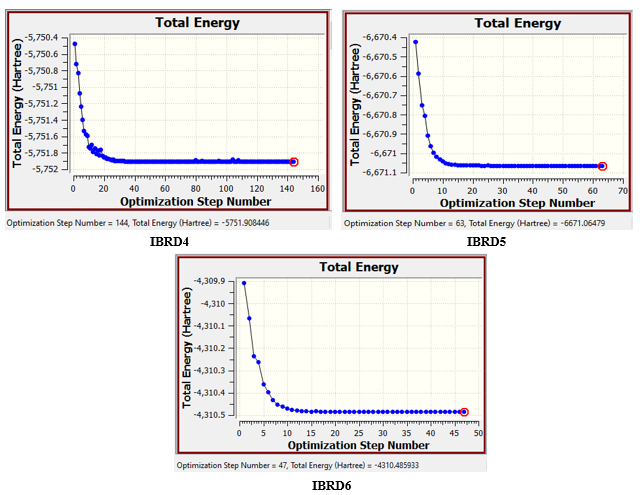


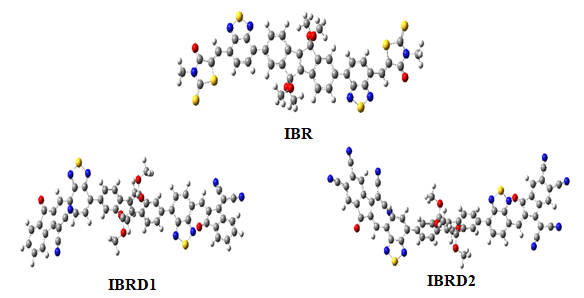


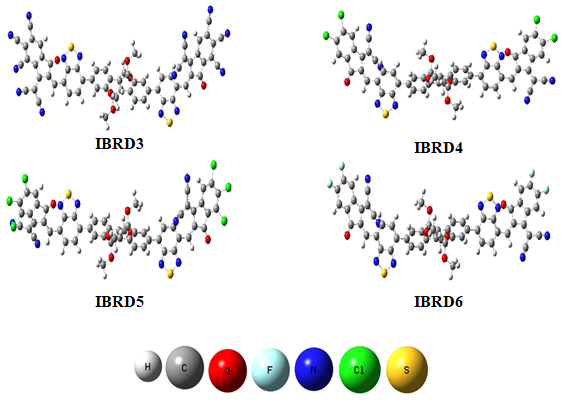


**Figure S1:** The optimization with frequency analyses based graphs as well as optimized structures of investigated acceptor chromophores are made with the help of GaussView 5 version. All out put files of entitled compounds were accomplished by Gaussian 09 version D.01 (<https://gaussian.com/g09citation/> )


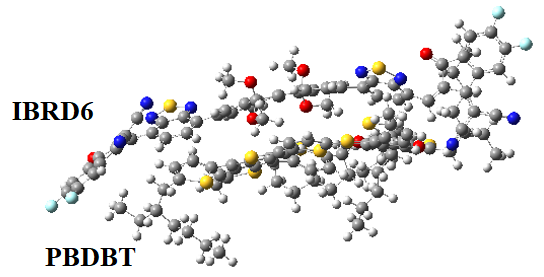


**(a)**


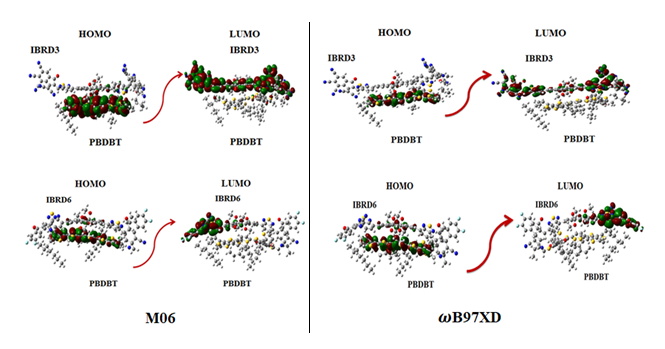


**(b)**

**Figure S2:** Optimized geometry of **PBDBT:IBRD6** (a) Comparative CT diagram of **PBDBT: IBRD3** and **PBDBT: IBRD6** at M06 and $\omega$B97XD functionals (b). are made with the help of GaussView 5 version ) and all out put files of entitled compounds were accomplished by Gaussian 09 version D.01 (<https://gaussian.com/g09citation/> )
